# Solar eclipse demonstrating the importance of photochemistry in new particle formation

**DOI:** 10.1038/srep45707

**Published:** 2017-04-04

**Authors:** Tuija Jokinen, Jenni Kontkanen, Katrianne Lehtipalo, Hanna E. Manninen, Juho Aalto, Albert Porcar-Castell, Olga Garmash, Tuomo Nieminen, Mikael Ehn, Juha Kangasluoma, Heikki Junninen, Janne Levula, Jonathan Duplissy, Lauri R. Ahonen, Pekka Rantala, Liine Heikkinen, Chao Yan, Mikko Sipilä, Douglas R. Worsnop, Jaana Bäck, Tuukka Petäjä, Veli-Matti Kerminen, Markku Kulmala

**Affiliations:** 1 Department of Physics, University of Helsinki, P.O. Box 64, FIN-00014 University of Helsinki, Finland; 2 Paul Scherrer Institute, 5232 Villigen – PSI, Switzerland; 3 Department of Forest Sciences, P.O. Box 27, FIN-00014 University of Helsinki, Finland; 4 Hyytiälä Forestry Field Station, Hyytiäläntie 124, FIN-35500 Korkeakoski, Finland; 5 Aerodyne Research Inc., Billerica, Massachusetts 01821, USA

## Abstract

Solar eclipses provide unique possibilities to investigate atmospheric processes, such as new particle formation (NPF), important to the global aerosol load and radiative balance. The temporary absence of solar radiation gives particular insight into different oxidation and clustering processes leading to NPF. This is crucial because our mechanistic understanding on how NPF is related to photochemistry is still rather limited. During a partial solar eclipse over Finland in 2015, we found that this phenomenon had prominent effects on atmospheric on-going NPF. During the eclipse, the sources of aerosol precursor gases, such as sulphuric acid and nitrogen- containing highly oxidised organic compounds, decreased considerably, which was followed by a reduced formation of small clusters and nanoparticles and thus termination of NPF. After the eclipse, aerosol precursor molecule concentrations recovered and re-initiated NPF. Our results provide direct evidence on the key role of the photochemical production of sulphuric acid and highly oxidized organic compounds in maintaining atmospheric NPF. Our results also explain the rare occurrence of this phenomenon under dark conditions, as well as its seemingly weak connection with atmospheric ions.

Solar eclipses have provided fundamental opportunities for scientific research, the most profound outcomes of which include the verification of Einstein’s general theory of relativity^1^ and characterization of the Sun’s atmosphere^2^. Sun-induced photochemical reactions sustain life on Earth and play essential roles in atmospheric chemistry. Quantifying the chain of atmospheric processes initiated by photochemical reactions is crucial for our understanding on aerosol and cloud formation and climate change.

New particle formation (NPF) produces around half of atmospheric cloud condensation nuclei, being important to the radiative balance and climate^3^. In most locations, observed NPF and subsequent particle growth take place during daytime only^4^. Simultaneously, photochemical cycles control the concentrations of reactive oxidants such as OH-radical and ozone. Clustering seems to be taking place also in the dark but these clusters are rarely growing^5^,^6^,^7^. Sulphuric acid, the principal driver of atmospheric NPF, is formed mainly by reaction of sulphur dioxide with OH-radicals, and to some extent also by its reaction with Criegee intermediates^8^,^9^,^10^. The newly formed particles are suggested to grow bigger in size mainly by condensation of low-volatile, highly-oxidized multifunctional compounds (HOMs) from terpene oxidation^11^,^12^,^13^,^14^. Some HOMs have extremely low volatilities (ELVOC) and they are able to condense onto sub-2 to 3 nm clusters, thus being responsible for the initial growth of fresh-formed particles and also a large part of secondary organic aerosol (SOA)^11^,^14^,^15^. Despite very high O:C ratios, not all HOMs are ELVOCs^16^, although the terms have been used interchangeably in previous studies (see SI for more details)^11^.

In the presence of NOx, also nitrogen containing HOMs (N-HOMs) are formed, and they have been observed during NPF events^17^,^18^,^19^, but the exact contribution of N-HOMs vs. HOMs that do not contain nitrogen on NPF is not known. In general, very little is known about the detailed connection between atmospheric photochemistry, production and composition of different low-volatility compounds, cluster formation and atmospheric NPF.

We measured aerosol precursor molecules, clusters and nanoparticles during a partial solar eclipse (81% of the sun blocked) on 20 March 2015 at SMEAR II-station, Finland^20^. Sudden changes in oxidant production during the eclipse gave us a unique opportunity to investigate the importance of photochemistry, especially OH-radical driven reactions, on the gas-to-particle conversion process. Similar boundary conditions are very difficult to find from any other kind of field measurement data. For example, daytime and night-time data differ from each other not only in terms of the strength of photochemistry, but also in terms of the chemical mixture of air pollutants and the presence of an additional oxidant (nitrate radical) during night time. Comparing data from clear-sky and cloudy days to each other would not allow isolating the effect of photochemistry either, as clear-sky and cloudy days tend to have different air mass properties. The closest analogy to a solar eclipse would be a transition between clear and cloudy skies. The problem with that data is that such transitions tend follow each other too rapidly to track the whole sequence of processes starting from atmospheric oxidation to the formation of growing nanoparticles.

## Results

**Figure 1:**
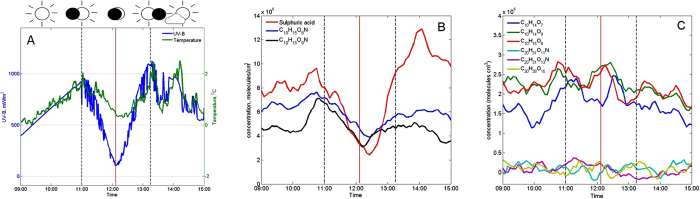
UV-B radiation (mW/m^2^), air temperature (°C) (**A**) and neutral aerosol precursor molecules measured on the eclipse day (**B**,**C**). The time series in panel B depicts CI-APi-TOF measurements of sulphuric acid and two N-HOMs (C_10_H_15_O_8_N, 339.068 Th and C_10_H_15_O_9_N, 355.063 Th) whose concentration decreased the most during the eclipse. Panel **C** shows other measured (N-)HOMs that stayed more constant during the eclipse: C_10_H_14_O_7_ (308.062 Th), C_10_H_14_O_9_ (340.052 Th) and C_10_H_15_O_8_ (325.065 Th), C_20_H_31_O_11_N (523.178 Th), C_20_H_31_O_13_N (555.168 Th) and C_20_H_28_O_15_ (570.131 Th). All mentioned m/z contain the mass of the charger ion, NO_3_^−^. Vertical red line: maximum phase, dashed black lines: beginning and end time of the eclipse.

The eclipse day was a sunny, mostly clear-sky NPF event day with light winds (2−3 m/s). During the eclipse, we observed a drop in the UV-B radiation intensity and ambient temperature (Fig. 1A), but no apparent change in either wind speed or direction. A drop similar to the UV-B radiation is collaterally expected in the OH-radical concentration (Fig. S1)^21^, for which we applied a proxy calculation in lack of direct measurements. Previous studies have shown a high correlation (>85%) between the OH-radical concentration and its primary production from ozone photolysis^22^,^23^, supporting the validity of our proxy calculation. After a notable decrease in the NO_x_ concentration prior to eclipse, concentrations of both NO and NO_x_ were low during the eclipse (Fig. S2). Also, the measured SO_2_ concentration remained low during the day (Fig. S2). The ozone concentration usually increases by about 10 ppb from morning to afternoon on springtime NPF event days at our measurement site^24^. The eclipse stalled this ozone increase momentarily, but did not cause any ozone decline as reported in earlier studies conducted in semi-polluted environments^22^,^25^. The lack of such decline in our measurements is probably related to the low NO_x_ concentration, making the ozone concentration less susceptible to its local photochemical production than in more polluted sites. We also observed an instant decrease in photochemical energy conversion rates in the forest and, subsequently, reductions in transpiration and CO_2_ exchange rates (Fig. S3). Monoterpene concentration decreased under detectable levels after the eclipse (Fig. S4).

The concentration of gaseous sulphuric acid (H_2_SO_4_) and two nitrogen containing HOMs (C_10_H_15_O_9_N, 355 Th and C_10_H_15_O_8_N, 339 Th, referred here after as N-HOM, m/z contains charger ion mass) measured by a nitrate based chemical ionization mass spectrometer (CI-APi-TOF)^26^ decreased considerably during the eclipse, indicating photochemical production pathways (Fig. 1B and S5). Local minima in sulphuric acid and concentrations of two major N-HOMs were observed 10–20 min after the minimum in UV-radiation (Fig. 1 and Figs S6–7), roughly consistent with the time scale over which any extremely low-volatile compound is expected to deplete from the gas phase due to its condensation onto pre-existing aerosol particles (the inverse of condensation sink, Fig. S8). The rest of measured HOMs and N-HOMs displayed highest concentrations well before the solar eclipse and did not decrease significantly during the eclipse (Fig. 1C and Fig. S9). The most plausible explanation for a HOM compound not to be affected by a solar eclipse is that its production in the gas phase is initiated, or maintained, by reactions involving ozone rather than photochemistry. Another possible explanation, at least for the HOMs that are not extremely low-volatile, is that they do not effectively condense onto pre-existing particles.

**Figure 2:**
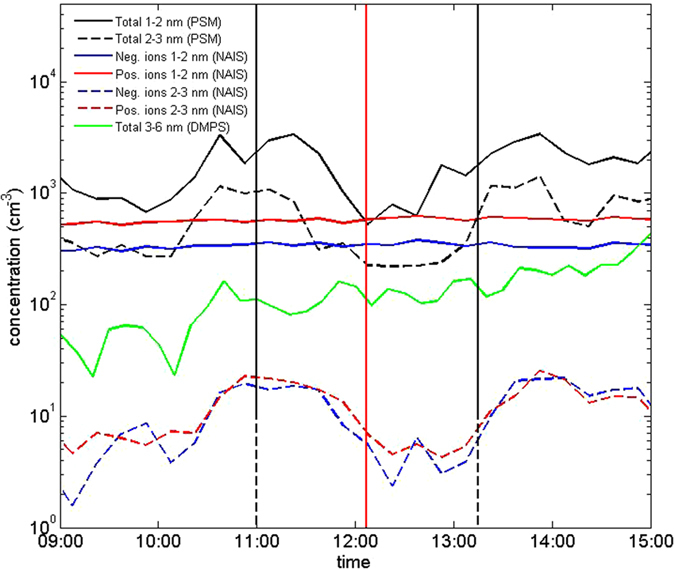
Measured concentrations of ions and nanoparticles during the eclipse. Solid black, red and blue lines in the time series depict the smallest 1–2 nm particles and dashed lines depict 2–3 nm particles. Black stands for the total concentration and blue and red for negative and positive ions, respectively. Green line shows the total concentration in the diameter range 3–6 nm. Vertical red line: maximum phase, dashed black lines: beginning and end time of the eclipse.

We measured ions and neutral particles using the particle size magnifier (PSM)^27^, neutral cluster and air ion spectrometer (NAIS)^28^ and differential mobility particle sizer (DMPS)^29^. Concentrations of 1−2 nm neutral particles and clusters dropped markedly during the eclipse (Fig. 2 and Fig. S10), confirming the strong association of the cluster formation with atmospheric photochemistry. Concentrations of <2 nm ions (cluster ions) were not affected by the eclipse as their primary production depends on ionizing radiation. Concentrations of 2−3 nm neutral particles and ions decreased by approximately the same factor, and their concentration minima were achieved a bit later than those of smaller particles. These features suggest that >2 nm particles originated mainly from the growth of sub-2 nm neutral clusters, and that ions in that size range originated from the charging of the neutral particles by cluster ions, consistent with earlier studies made at SMEAR II^17^,^30^. The concentration of 3–6 nm particles changed little during the eclipse, indicating that their formation rate was suppressed compared to regular NPF days at SMEAR II when their concentration usually increases rapidly around noon^31^,^32^. We did not observe any systematic change in the submicron aerosol mass concentration, or its bulk chemical composition, during the eclipse (Fig. S11).

**Figure 3:**
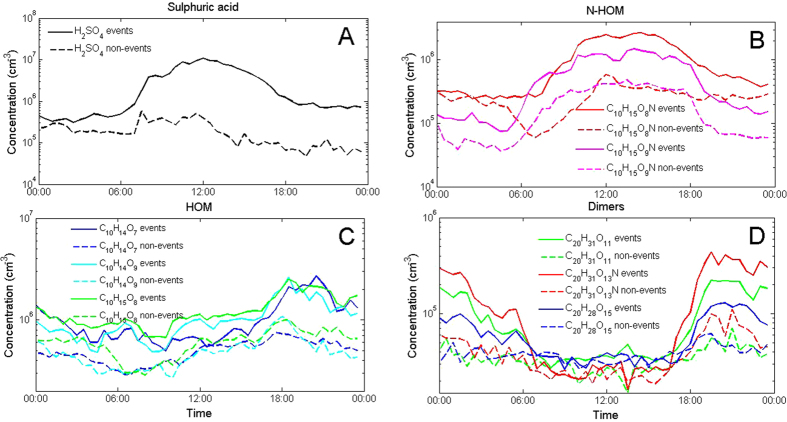
Diurnal behavior of sulphuric acid (**A**), nitrogen containing N-HOM (**B**), HOM in the monomer range (**C**) and HOM dimers (**D**) measured with the CI-APi-TOF at the SMEAR II-station in the spring 2011. Solid lines represent event days and dashed lines non-event days. Compounds in panel B are identified as C_10_H_15_O_8_N (339.068 Th) and C_10_H_15_O_9_N (355.063 Th) and in panel C as C_10_H_14_O_7_ (308.062 Th), C_10_H_14_O_9_ (340.052 Th) and C_10_H_15_O_8_ (325.065 Th). The compounds panel D are suggested to be C_20_H_31_O_11_N (523.178 Th), C_20_H_31_O_13_N (555.168 Th) and C_20_H_28_O_15_ (570.131 Th), but it is possible that other highly oxidized molecules could also contribute to these signals. All mentioned m/z contain the mass of the charger ion, NO_3_^−^.

Long-term observations at the SMEAR II-station show, on average, higher concentrations of sulphuric acid, N-HOM and several other HOM monomers during NPF event days compared with non-event days (Fig. 3). During the eclipse, the formation rate of 1.5 nm particles, *J*_1.5_, was positively correlated with both sulphuric acid and N-HOM concentration, but not with the concentrations of other HOMs (Figs S12–15). Furthermore, *J*_1.5_ was positively correlated with the product of the H_2_SO_4_ concentration and concentration of any of the major HOMs. Correlations with the formation rate of 2 nm particles, *J*_2_, were quantitatively similar but clearly weaker compared with *J*_1.5_ (Figs S16–18). While these observations do not proof any causality between NPF and sulfuric acid or (N-)HOMs, they definitely support the idea that both sulfuric acid and some subset of (N-)HOMs at large enough concentrations are needed for active NPF at this site.

During the recovery from the solar eclipse, sulphuric acid concentration increased rapidly (Fig. 1B), consistent with its UV-driven production during daytime (Figs S19 and S20). Concentrations of N-HOMs increased at a slower rate (Fig. 1B), since their production route requires the initiation of NO_2_ photolysis, VOC emissions and their oxidation to RO_2_ radicals via either OH-radical reactions or ozonolysis (Fig. S21). Concentrations of sub-3 nm clusters and particles recovered somewhat later than sulphuric acid and N-HOM, as one would expect if these compounds were important precursors for small clusters. The observed growth rate of 2–3 nm particles was ~1 nm/h during the recovery from eclipse (Fig. S22). While our data do not reveal which compounds actually contributed to the particle growth, it is interesting to note that sulphuric acid together with the two major N-HOM compounds had high enough concentrations so that their concomitant, irreversible condensation onto sub-3 nm particles could explain the observed growth.

In summary, we have shown that the decrease in UV-radiation during the solar eclipse decreases the source rates of sulphuric acid and some (N-)HOMs, which further leads to a decrease in clustering and initial growth of aerosol particles. Since the condensation sink is hardly affected by the eclipse, low-volatile vapours continue condensing and small clusters coagulating onto existing aerosol particles. Thus, the gas-phase concentrations of the low-volatile vapour originating mainly from photochemistry and the number concentration of small clusters made of these vapours decrease notably. During the recovery phase of the eclipse, OH-radical, sulphuric acid, (N-)HOM and cluster concentrations recover and re-initialize NPF.

Our observations might explain why the frequently-observed night-time sub-3 nm clusters do not grow into larger aerosol particles. We conclude that when ozonolysis is the prominent formation route of condensing vapours (like HOMs at night-time, Fig. 3), the resulting ozonolysis products alone cannot grow these sub-3 nm clusters further into new aerosol particles in a boreal forest atmosphere at least in the observed concentration levels. Thus, extremely low-volatile reaction products from the OH-radical oxidation, especially sulphuric acid and a sub-set of (N-)HOMs, are the key compounds for cluster growth and observing NPF. Furthermore, the build-up of HOMs and their dimers in the morning, apparent prior to the eclipse (Figs 3 and S9) and during regular NPF event days compared with non-event days, may boost the particle growth.

We had a unique possibility to investigate atmospheric chemistry and clustering related to NPF during a partial solar eclipse at the SMEAR II-station using state-of-the-art instrumentation. Our results highlight the necessity of photochemistry in aerosol precursor formation and provide a plausible explanation of why NPF is observed almost solely during daytime in continental boundary layers. Our results confirm the crucial role of sulphuric acid in maintaining atmospheric NPF and show further that extremely low volatile organic compounds, potentially important to the initial steps of atmospheric NPF, are formed not only by ozone oxidation but also through photochemical reactions. Our results show that neutral pathways dominate the initial clustering and growth, at least at our measurement site. Such consistent observations, from the oxidation of precursor vapours to nanoparticles, have previously been achieved only in controlled laboratory experiments. Our results are an important step toward understanding the connections between atmospheric oxidation, NPF and secondary aerosol formation, which is needed for quantifying the complex interplay between future anthropogenic activities, air pollution and changing global climate.

## Methods

All measurements were conducted at the SMEAR II-station, located in Hyytiälä (61°51′N, 24°17′E, 181 m a.s.l.), Southern Finland^20^. The main instrumentation was a mass spectrometer, a particle size magnifier and an ion mobility spectrometer. The mass spectrometric measurements were conducted with a CI-APi-TOF with nitrate based chemical ionization scheme in the negative ion mode^26^ for the detection of aerosol precursor molecules and clusters such as sulphuric acid and highly oxidized organic compounds. Low volatile compounds were detected after a proton transfer or clustering with the charger ions. The number concentration of freshly formed 1–3 nm particles and clusters were measured by a PSM^27^ that used diethylene glycol to activate and grow particles to 90 nm. After that, particles grow to detectable sizes by condensation of butanol inside a condensation particle counter. Neutral cluster and Air Ion Spectrometer (NAIS)^33^,^34^ is an ion mobility spectrometer that was used to detect the size distributions of ions between 0.8 nm and 42 nm (mobility equivalent diameter) and total particles, i.e. neutral and charged, between ~2 nm and 42 nm. More detailed information of instrumentation and other used methods can be found in the Supplementary material.

## Additional Information

**How to cite this article:** Jokinen, T. *et al*. Solar eclipse demonstrating the importance of photochemistry in new particle formation. *Sci. Rep.* **7**, 45707; doi: 10.1038/srep45707 (2017).

**Publisher's note:** Springer Nature remains neutral with regard to jurisdictional claims in published maps and institutional affiliations.

## Supplementary Material

Supplementary Material
