# Supplementary material for: Solar eclipse demonstrating the importance of photochemistry in new particle formation
Source: Sci Rep. 2017 Apr 4;7:45707. doi: 10.1038/srep45707 (PMC5379550; doi:10.1038/srep45707)
Supplement: Supplementary Material [file srep45707-s1.pdf]

Supplementary material

Title: Solar eclipse demonstrating the importance of photochemistry in new particle formation

Authors: Tuija Jokinen*, Jenni Kontkanen, Katrianne Lehtipalo, Hanna E. Manninen, Juho Aalto, Albert Porcar-Castell, Olga Garmash, Tuomo Nieminen, Mikael Ehn, Juha Kangasluoma, Heikki Junninen, Janne Levula, Jonathan Duplissy, Lauri R. Ahonen, Pekka Rantala, Liine Heikkinen, Chao Yan, Mikko Sipilä, Douglas R. Worsnop, Jaana Bäck, Tuukka Petäjä, Veli-Matti Kerminen and Markku Kulmala

* correspondence to tuija.jokinen@helsinki.fi

SMEAR Station

SMEAR II (Station for Measuring Ecosystem – Atmosphere Relations) station is part of four research stations established in Finland to measure the relationship of atmosphere and forest in boreal climate zone (*1*). SMEAR II-station is located in Hyytiälä (61°51′ N, 24°17′E, 181 m a.s.l.), Southern Finland, in the middle of a boreal forest, surrounded by a fairly homogeneous Scots pine (*Pinus sylvestris*) population. Areas in the North and North-East are surrounded by small lakes and wetlands.

The station was opened in 1995 and has been providing research facilities to many researchers ever since. Hyytiälä has a comprehensive measurement set up for the observations in the ecosystem-atmosphere interactions, most of which are presented in this article. Some observations, including ozone and NO, are made utilizing the mast of 127 m. Measurements can be done at seven different heights of the mast with high time resolution up to 2 minutes. The canopy height is approximately 18 m. Some measurements are conducted below canopy and some above utilizing the mast or the new 35 m walk up tower that accommodates instruments for aerosol, gas and cluster measurements.

Hyytiälä is located about 60 km North-East from the third biggest city of Finland, Tampere. Most pollution transported to the measurement site originates either from Tampere area or from a saw mill located in Korkeakoski, about 10 km South-East from SMEARII. Detailed information of the SMEARII-station can be read in Hari and Kulmala (2005) (*1*). Detailed description of the land use and vegetation surrounding Hyytiälä is described in details by Haapanala et al. (2007) (*2*), Ilvesniemi et al. (2009) (*3*) and Williams et al. (2011) (*4*).

Main Instrumentation and methods

CI-APi-TOF

The Chemical Ionization Atmospheric Pressure interface Time Of Flight (CI-APi-TOF) mass spectrometer was measuring above canopy in the 35 m walk up tower. The used mass spectrometer was originally developed for precise sulphuric acid measurements (*5*) but nowadays the development of the technique utilizes high resolution time-of-flight mass spectrometers and is able to detect molecular sulphuric acid (*6*), sulphuric acid containing clusters (*7*) and highly oxidized organic molecules (*8*, *9*). HOMs measured with the CI-APi-TOF are estimated (calculated) to have low vapour pressures and this work reports exactly the same compound groups detected in previous laboratory experiments and publications, where compounds from $C_{10}H_{16}O_7$ and higher oxygen numbers are classified as extremely low-volatile organic compounds (ELVOC) in 273 K temperatures (*10*, *11*). During the eclipse day 20 Mar 2016, temperature was around 273±2 K. Measurements in Fig.3 were measured during a spring campaign between 14 Mar and 11 Apr 2011 from close to ground level.

In previous studies (e.g. (*11*) all highly oxidized molecules measured by the CI-APi-TOF were somewhat incorrectly termed as ELVOCs. More recent studies, including both computational (*12*) and in laboratory (e.g. (*13*) suggest that many of the HOMs found by Ehn et al. are LVOCs rather than ELVOCs.

The instrument was run in the negative ion mode and with an active chemical ionization method using charged HNO3 (→ NO3-) as a reagent ion. The sample is introduced via ~60 cm ¾" tube with a flow rate of 10 slpm (standard L/minute). The sample is surrounded with a sheath flow containing the reagent ions which are then guided to meet the sample using an electric field. Sample is charged either with proton transfer from the acidic sample molecule (AH) to the reagent ions (B) via

$AH + B^- \rightarrow A^- + BH$

or via clustering

$AH + B^- \rightarrow AHB^-$

Using chemical ionization is a highly selective method for detection of low-volatility aerosol precursors and molecules from ambient air. Detailed information of the CI-APi-TOF can be obtained from Jokinen et al. 2012(*6*).

The concentration of sample molecules is calculated as following:

$$[sample]_{neutral} = \frac{sample^{-} + sample{\cdot}NO_3^-}{\sum \text{reagent ions}} x\ C \quad (1)$$

,where $[\text{sample}]_{\text{neutral}}$ is the calculated concentration (in molecules/cm^3) of the neutral measured compound e.g. sulphuric acid, sample^- is the measured signal of sample chemically charged sample charged via proton transfer, sample · NO_3^- is the measured charged sample signal formed via clustering and C is a calibration factor. Reagent ions in the equation are the sum of signals from NO_3^-, $HNO_3NO_3^-$ and $(HNO_3)_2NO_3^-$.

Calibration is done using a sulphuric acid generator described by Kürten et al., (2012) (*14*). Calibration factor determined for these measurements using the sulphuric acid generator was 2.2e10 molecules/cm^3.

Particle Size Magnifier (PSM)

The Airmodus nano Condensation Nuclei Counter (nCNC)(*15*) is a mixing-type condensation particle counter (CPC) consisting of a Particle Size Magnifier (PSM) and a conventional CPC. Inside the PSM, diethylene glycol is used to activate and grow particles to 90 nm. After that, particles grow to detectable sizes by condensation of butanol inside the CPC. In this study, the cut-off size of the PSM was varied between approximately 1 and 3 nm (electrical mobility equivalent diameter) by altering the mixing ratio of the sample and saturator flow rates. Changing the supersaturation from the smallest to the highest value takes two minutes. The conversion from the PSM saturator flow rate to the cut-off size contains uncertainties related to the particle composition (*16*). Mixture compounds of sulfuric acid and nitrogen containing HOMs are observed to nucleate in Hyytiälä (*17*), and the range of supersaturations inside the PSM are adjusted accordingly. The data inversion was conducted by assuming Gaussian-shaped kernel functions and the concentration of clusters in three sub-3 nm size bin was obtained(*18*). Uncertainties in the inverted size bins reflect the small variations in the PSM cut-off sizes due to ambient water vapor concentration variations, and uncertainties in the particle activation due to unknown exact particle composition and resulting cut-off sizes. The data was inverted to size bins of < 1.5 nm, 1.5 – 2 nm and 2 – 3 nm +- 0.4 nm with time resolution of 10 min. Note that in this article, the whole nCNC-system is referred to as the PSM, as is commonly done in the literature.

Neutral cluster and Air Ion Spectrometer (NAIS)

Neutral cluster and Air Ion Spectrometer (NAIS) (*19*, *20*) is an ion mobility spectrometer measuring the size distributions of ions between 0.8 nm and 42 nm (mobility equivalent diameter) and total particles, i.e. neutral and charged, between ~2 nm and 42 nm. When total particles are measured, the sample aerosol is charged with a corona charger. Two cylindrical differential mobility analysers classify positive and negative ions based on their electrical mobility and 21 electrometers on the outer cylinder detect the ions. High sample flow rate (54 l/min) is used to minimize the diffusion losses. Time resolution of the NAIS data is 3 minutes.

Aerosol Chemical Speciation Monitor (ACSM)

The ACSM (*21*) measures the non refractory aerosol chemical composition, e.g. the mass concentration of organics, sulfates, nitrates, ammonium and chlorides within the vacuum aerodynamic diameter range of 75 – 650 nanometers (*22*). The sample air is focused with aerodynamic lenses, flash vaporized in 600 ˚C and electron impact ionized (EI) with 70 electronvolts. The mass analyzer is a residual gas analyzer (RGA) type quadrupole.

Growth rates

The growth rate of sub-3nm clusters was determined using the NAIS ion data by fitting a Gaussian distribution to the concentration time series at a certain size to determine the moment of maximum concentration. The growth rate was obtained by a linear fit to the moments of maximum concentrations and the corresponding geometric mean diameters of the particles. For the comparison of this method to other growth rate methods, see Yli-Juuti et al. (2011) (*23*).

Formation rates

The cluster formation rates at 1.5 nm ($J_{1.5}$), and at 2 nm (J_2) were calculated from PSM data according to Kulmala et al. (2012) (*19*):

$$J_{Dp} = \frac{dN_{Dp}}{dt} + CoagS_{Dp} \cdot N_{Dp} + \frac{GR_{Dp}}{\Delta Dp} \cdot N_{Dp} .$$

Here N_{Dp} is the cluster concentration, $CoagS_{Dp}$ refers to the coagulation sink, and GR_{Dp} is the cluster growth rate. Thus, Eq. (2) takes into account the losses of clusters due to coagulation and growth out of the studied size range.

The formation rates of positive and negative ions at 2 nm ($J_2^{\pm}$) were calculated from NAIS ion mode data (*19*):

$$J_2^{\pm} = \frac{dN_{Dp}^{\pm}}{dt} + CoagS_{Dp} \cdot N_{Dp}^{\pm} + \frac{GR_{Dp}}{\Delta Dp} \cdot N_{Dp}^{\pm} + \alpha \cdot N_{Dp}^{\pm} \cdot N_{<Dp}^{\mp} - \beta \cdot N_{Dp} \cdot N_{<Dp}^{\pm}. \quad (3)$$

In addition to the losses due to coagulation and growth, the loss of ions due to ion-ion recombination and the source of ions due to charging of neutral clusters were taken into account. The ion-ion recombination coefficient, α, and ion-neutral attachment coefficient, β , were assumed to equal $1.6 \cdot 10^{-6}$ cm^3 s^{-1} and $0.01 \cdot 10^{-6}$ cm^3 s^{-1}, respectively.

Proxy for OH

The proxy for the concentration of hydroxyl radical (OH) was calculated by scaling the measured UV-B radiation with empirically derived factors according to Petäjä et al. (2009) (*24*):

$$[OH]_{proxy} = \left(\frac{8.4\times10^{-7}}{8.6\times10^{-10}} UVB^{0.32}\right)^{1.92} \quad (4)$$

Simple model calculations

Simple model calculations were conducted by assuming that the time evolution of the concentration of sulfuric acid and N-HOMs during the eclipse can be expressed as:

$$\frac{dC}{dt} = A \times UVB - CS \times C \quad (5)$$

Here UVB is measured UV-B radiation and CS is the condensation sink.

The value for the coefficient A was obtained by assuming that in the beginning of the eclipse the vapor production rate equals the loss rate of vapor by condensation:

$$A \times UVB_0 = CS_0 \times C_0.$$

For sulfuric acid we also tested another approach, where the dependence of vapor production rate on SO_2 concentation was taken into account. In this case, the time evolution of sulfuric acid concentration is obtained from:

$$\frac{dC}{dt} = B \times [SO_2] \times UVB - CS \times C \quad (6)$$

Correspondingly, the value for the coefficient B was calculated from:

$$B \times [SO_2]_0 \times UVB_0 = CS_0 \times C_0.$$

Furthermore, to see the effect of CS on the modelled concentration, we performed additional simulations where CS was set to zero after the minimum of the measured sulfuric acid concentration.

Supporting results from the simple model

The sulphuric acid and N-HOM concentrations as a function of UV–B intensity show that the values measured during the decreasing phase of UV-radiation were somewhat higher than those during the increasing phase of UV-radiation as seen in Figure S5 and S6. This feature is consistent with their assumed photochemical production pathways and their removal processes. The decrease in the concentrations of low-volatile vapours during the eclipse can be explained by their condensation onto existing aerosol particles. Throughout the eclipse, the condensation sink (CS) was around 0.0004 s^{-1} and stayed almost constant (Figure S9). However, when modelling the production of condensing vapours during the recovery from the solar eclipse, we see that the pre-existing aerosols do not accommodate sulphuric acid as effectively as possible (Figure S11), especially when compared with a day without a solar eclipse (see Figure S11). The recovery of the main N-HOM concentration from the solar eclipse is simulated surprisingly well with the simple model, indicating its irreversible condensation onto the measured pre-existing particles.

Shoot-level gas exchange and electron transport rate measurements

Gas-exchange (CO_2, H_2O, VOCs) of Scots pine shoots were measured using four dynamic branch enclosures with an automated sampling and gas-analyser setup. The enclosures were installed to unshaded top-canopy conditions and there were 48-72 closures per day for each enclosure. The approach is described in detail by Hari et al. 1999 (*25*) and Aalto et al. 2015 (*26*).

Photosynthetic energy conversion and electron transport rate were measured using a PAM chlorophjyll fluorescence monitoring system (Monitoring PAM, Walz GmbH, Germany). The system included five fluorometers distributed across three Scots pine trees (3 in top canopy and 2 in low canopy needles). Measurements were carried out at a frequency of 30 minutes. At each measuring point PAR, air temperature, needle-level instantaneous fluorescence yield (F’) and maximal fluorescence yield (F’m) are retrieved. The rate of electron transport in photosystem II (PSII) is estimated as:

$$ETR = PAR\ A\ a_{II}\ \frac{F'_m - F'}{F'_m} \quad (7)$$

Where A is leaf absorptance (equal to 0.82 for our pine needles), a_{II} is the relative absorption cross section of PSII (assumed to be 0.5) (*27*). Further details on these measurements can be found in Porcar-Castell et al. 2008 (*28*) and Porcar-Castell 2011 (*29*).

References

1. P. Hari, M. Kulmala, Station for Measuring Ecosystem-Atmosphere Relations (SMEAR II). *Boreal Environ. Res.* **10**, 315–322 (2005).
2. S. Haapanala *et al.*, Boundary layer concentrations and landscape scale emissions of volatile organic compounds in early spring. *Atmos. Chem. Phys.* **7**, 1869–1878 (2007).
3. H. Ilvesniemi *et al.*, Long-term measurements of the carbon balance of a boreal Scots pine dominated forest ecosystem. *Boreal Environ. Res.* **14**, 731–753 (2009).
4. J. Williams *et al.*, The summertime Boreal forest field measurement intensive (HUMPPA-COPEC-2010): an overview of meteorological and chemical influences. *Atmos. Chem. Phys.* **11**, 10599–10618 (2011).
5. F. L. Eisele, D. J. Tanner, Measurement of the gas phase concentration of H 2 SO 4 and methane sulfonic acid and estimates of H 2 SO 4 production and loss in the atmosphere. *J. Geophys. Res.* **98**, 9001 (1993).
6. T. Jokinen *et al.*, Atmospheric sulphuric acid and neutral cluster measurements using CI-APi-TOF. *Atmos. Chem. Phys.* **12**, 4117–4125 (2012).
7. A. Kürten *et al.*, Neutral molecular cluster formation of sulfuric acid–dimethylamine observed in real time under atmospheric conditions. *Proc. Natl. Acad. Sci.* **111**, 15019–15024 (2014).
8. S. Schobesberger *et al.*, Molecular understanding of atmospheric particle formation from sulfuric acid and large oxidized organic molecules. *Proc. Natl. Acad. Sci. U. S. A.* **110**, 17223–8 (2013).
9. F. Riccobono *et al.*, Oxidation Products of Biogenic Emissions Contribute to Nucleation of Atmospheric Particles. *Science (80-.).* **344**, 717–721 (2014).
10. T. Jokinen *et al.*, Production of extremely low volatile organic compounds from biogenic emissions: Measured yields and atmospheric implications. *Proc. Natl. Acad. Sci. U. S. A.* **112**, 7123–7128 (2015).
11. M. Ehn *et al.*, A large source of low-volatility secondary organic aerosol. *Nature*. **506**, 476–9 (2014).
12. T. Kurtén *et al.*, α-pinene Autoxidation Products May Not Have Extremely Low Saturation Vapor Pressures Despite High O:C Ratios. *J. Phys. Chem. A*. **120**, 2569–2582 (2016).
13. J. Tröstl *et al.*, The role of low-volatility organic compounds in initial particle growth in the atmosphere. *Nature*. **533**, 527–531 (2016).
14. A. Kürten, L. Rondo, S. Ehrhart, J. Curtius, Calibration of a chemical ionization mass spectrometer for the measurement of gaseous sulfuric acid. *J. Phys. Chem. A*. **116**, 6375–6386 (2012).
15. J. Vanhanen *et al.*, Particle Size Magnifier for Nano-CN Detection. *Aerosol Sci. Technol.* **45**, 533–542 (2011).
16. J. Kangasluoma *et al.*, Sub-3 nm particle size and composition dependent response of a nano-CPC battery. *Atmos. Meas. Tech.* **7**, 689–700 (2014).
17. M. Kulmala *et al.*, Direct observations of atmospheric aerosol nucleation. *Science*. **339**, 943–6 (2013).
18. K. Lehtipalo *et al.*, Methods for determining particle size distribution and growth rates between 1 and 3 nm using the Particle Size Magnifier. *Boreal Env. Res.* **19 (suppl.**, 215–236 (2014).

19. M. Kulmala *et al.*, Measurement of the nucleation of atmospheric aerosol particles. *Nat. Protoc.* **7**, 1651–67 (2012).

20. S. Mirme, A. Mirme, The mathematical principles and design of the NAIS – a spectrometer for the measurement of cluster ion and nanometer aerosol size distributions. *Atmos. Meas. Tech.* **6**, 1061–1071 (2013).

21. N. L. Ng *et al.*, An Aerosol Chemical Speciation Monitor (ACSM) for Routine Monitoring of the Composition and Mass Concentrations of Ambient Aerosol. *Aerosol Sci. Technol.* **45**, 780–794 (2011).

22. P. S. K. Liu *et al.*, Transmission Efficiency of an Aerodynamic Focusing Lens System: Comparison of Model Calculations and Laboratory Measurements for the Aerodyne Aerosol Mass Spectrometer. *Aerosol Sci. Technol.* **41**, 721–733 (2007).

23. T. Yli-Juuti *et al.*, Growth rates of nucleation mode particles in Hyytiälä during 2003−2009: variation with particle size, season, data analysis method and ambient conditions. *Atmos. Chem. Phys.* **11**, 12865–12886 (2011).

24. T. Petäjä *et al.*, Sulfuric acid and OH concentrations in a boreal forest site. *Atmos. Chem. Phys.* **9**, 7435–7448 (2009).

25. P. Hari *et al.*, An improvement of the method for calibrating measurements of photosynthetic CO2 flux. *Plant, Cell Environ.* **22**, 1297–1301 (1999).

26. J. Aalto *et al.*, Onset of photosynthesis in spring speeds up monoterpene synthesis and leads to emission bursts. *Plant. Cell Environ.* **38**, 2299–312 (2015).

27. K. Maxwell, K. N. Johnson, Chlorophyll fluorescence--a practical guide. *J. Exp. Bot.* **51**, 659–668 (2000).

28. A. Porcar-Castell, E. Pfündel, J. F. J. Korhonen, E. Juurola, A new monitoring PAM fluorometer (MONI-PAM) to study the short- and long-term acclimation of photosystem II in field conditions. *Photosynth. Res.* **96**, 173–9 (2008).

29. A. Porcar-Castell, A high-resolution portrait of the annual dynamics of photochemical and non-photochemical quenching in needles of Pinus sylvestris. *Physiol. Plant.* **143**, 139–53 (2011).

30. J. P. Abram, D. J. Creasey, D. E. Heard, J. D. Lee, M. J. Pilling, Hydroxyl radical and ozone measurements in England during the solar eclipse of 11 August 1999. *Geophys. Res. Lett.* **27**, 3437–3440 (2000).

31. R. Mauldin *et al.*, South Pole Antarctica observations and modeling results: New insights on HOx radical and sulfur chemistry. *Atmos. Environ.* **44** (2010), pp. 572–581.

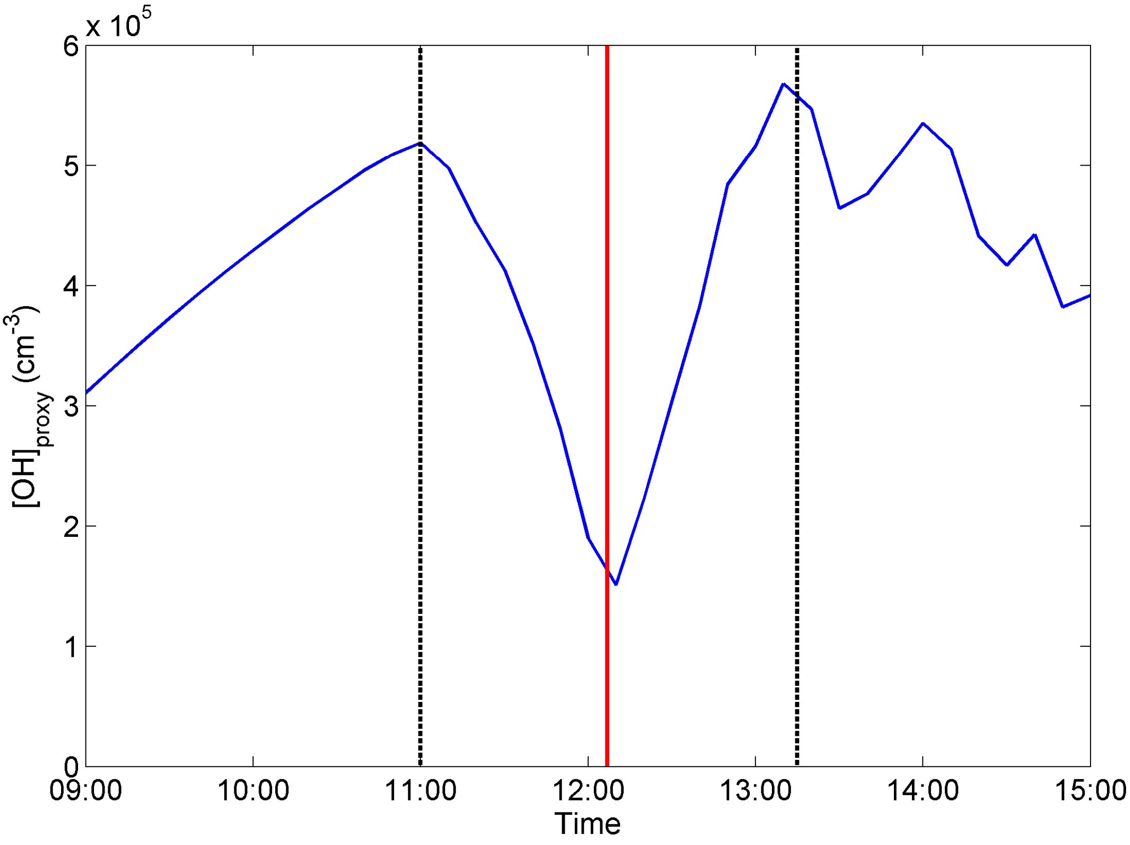

Figure S1: Proxy for the concentration of hydroxyl radical (OH) calculated according to Petäjä et al. (2009). . Proxy calculation was used because OH-radicals are not measured continuously and the correlation to primary production from ozone photolysis in previous studies is very high (>85 %)(*30, 31*). Verticaled line: maximum phase, dashed black lines: beginning and end time of the eclipse.

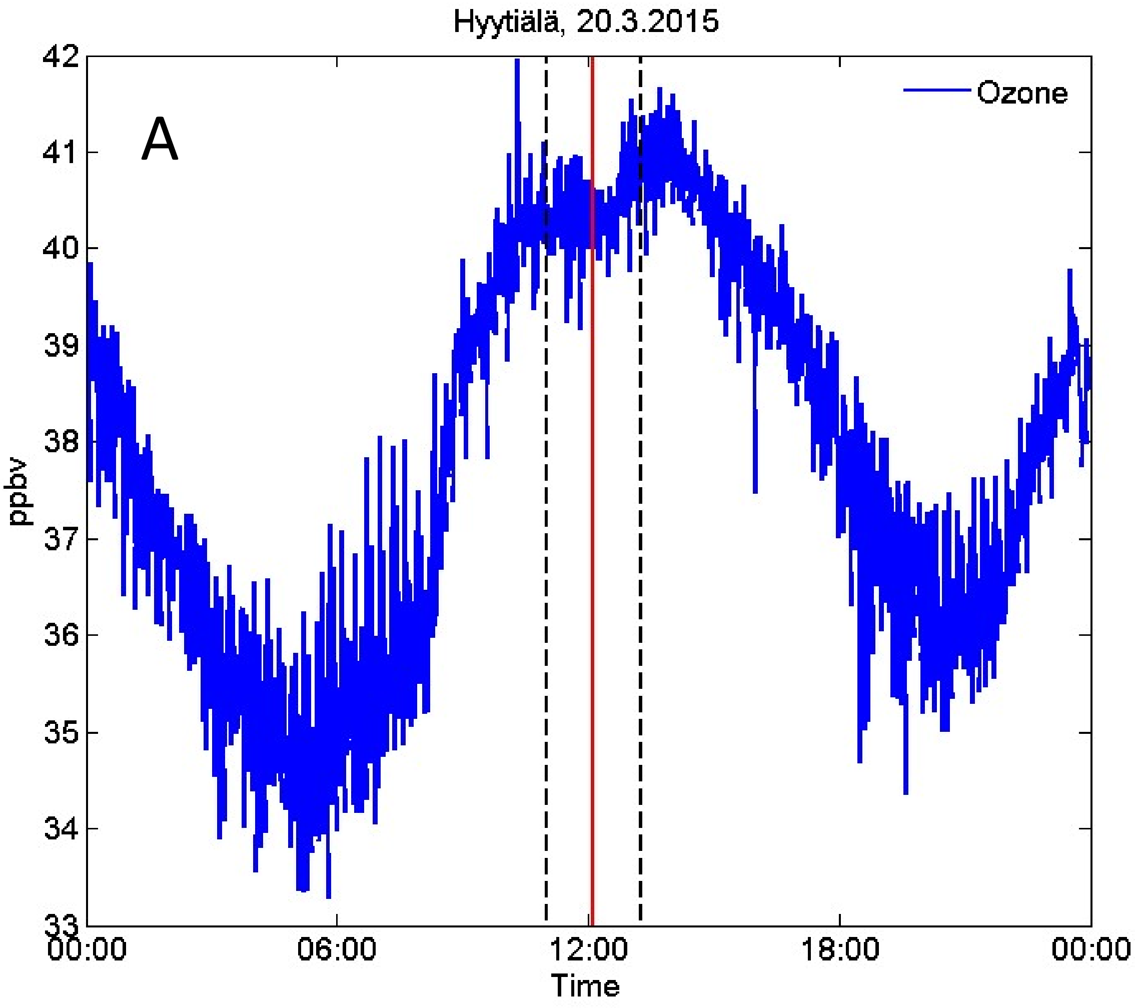
Hyytiälä, 20.3.2015
A
Ozone
ppbv
42
41
40
39
38
37
36
35
34
33
00:00
06:00
12:00
18:00
00:00
Time

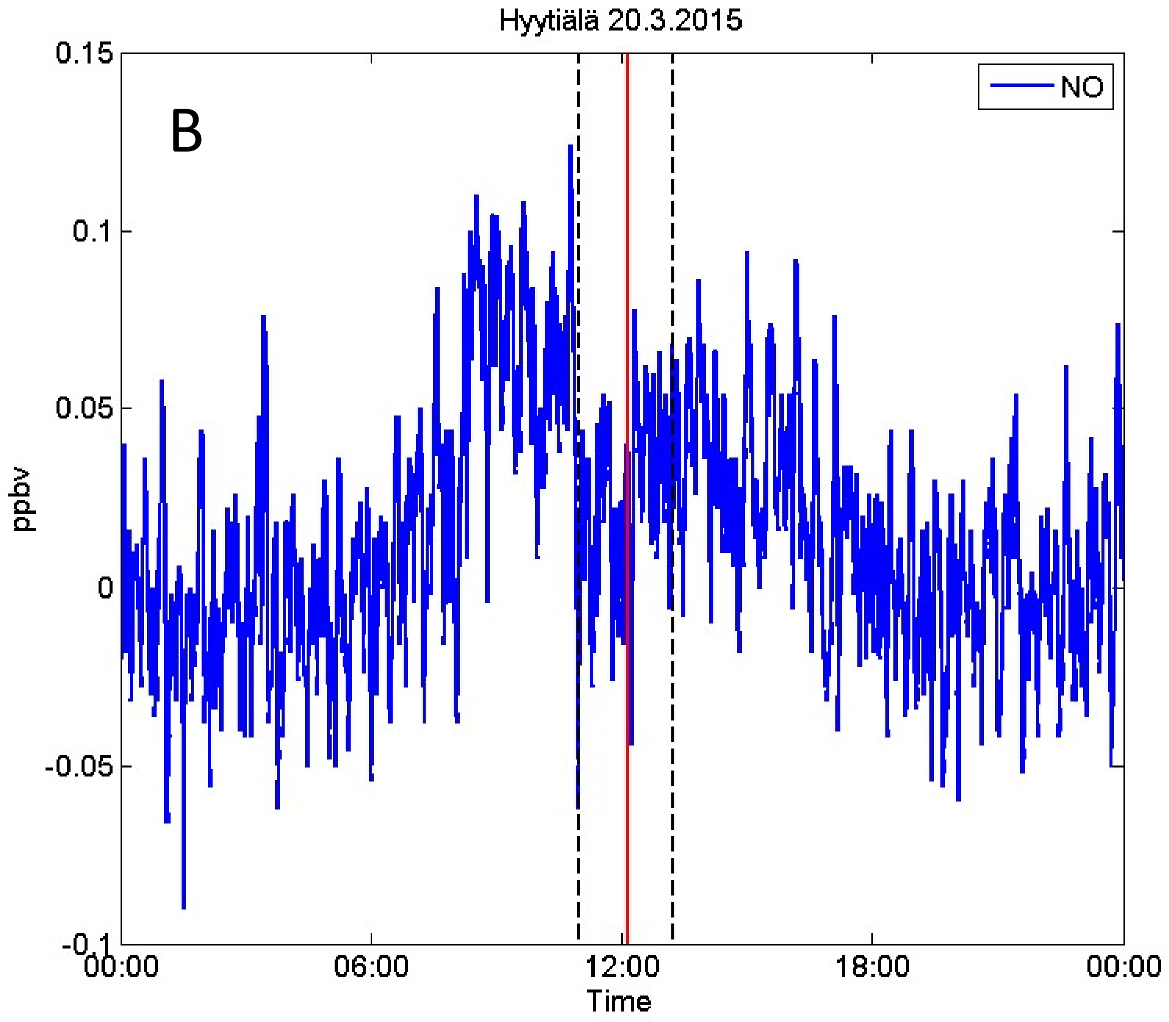
Hyytiälä 20.3.2015
B
NO
0.15
0.1
0.05
0
-0.05
-0.1
ppbv
00:00
06:00
12:00
18:00
00:00
Time

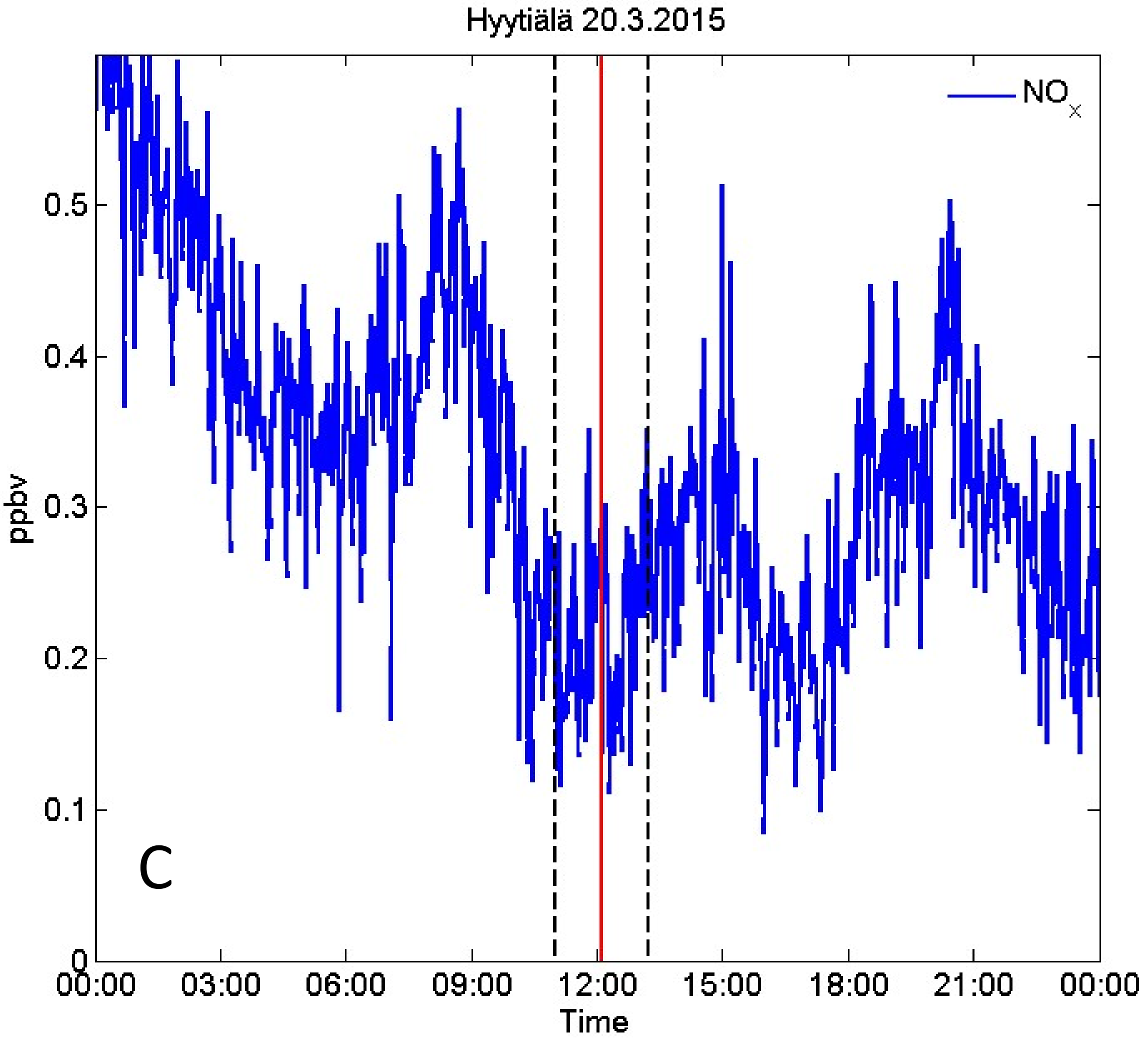
Hyytiälä 20.3.2015
NO$_x$
ppbv
0.5
0.4
0.3
0.2
0.1
0
C
00:00
03:00
06:00
09:00
12:00
15:00
18:00
21:00
00:00
Time

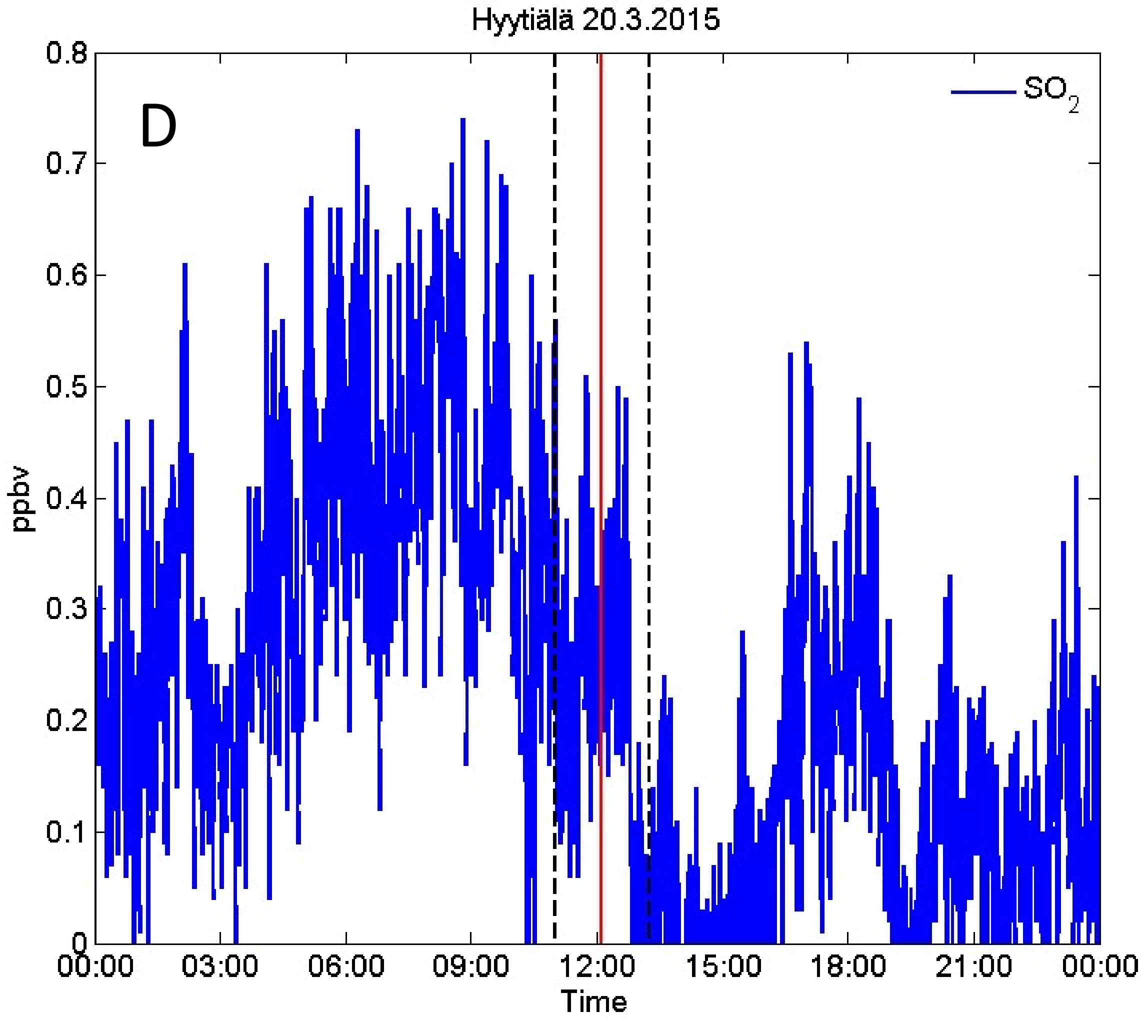

Figure S2: Trace gas concentrations during the solar eclipse day. Ozone (A) and NO (B) and NOx (C) concentrations are 2-minute averages over measurements from seven different heights from the mast (4.2 – 125 m) and SO_2 (D) is measured at one level (16.8 m), and also averaged for 2-minute datasets. Red line: maximum phase, dashed black lines: beginning and end time of the eclipse.

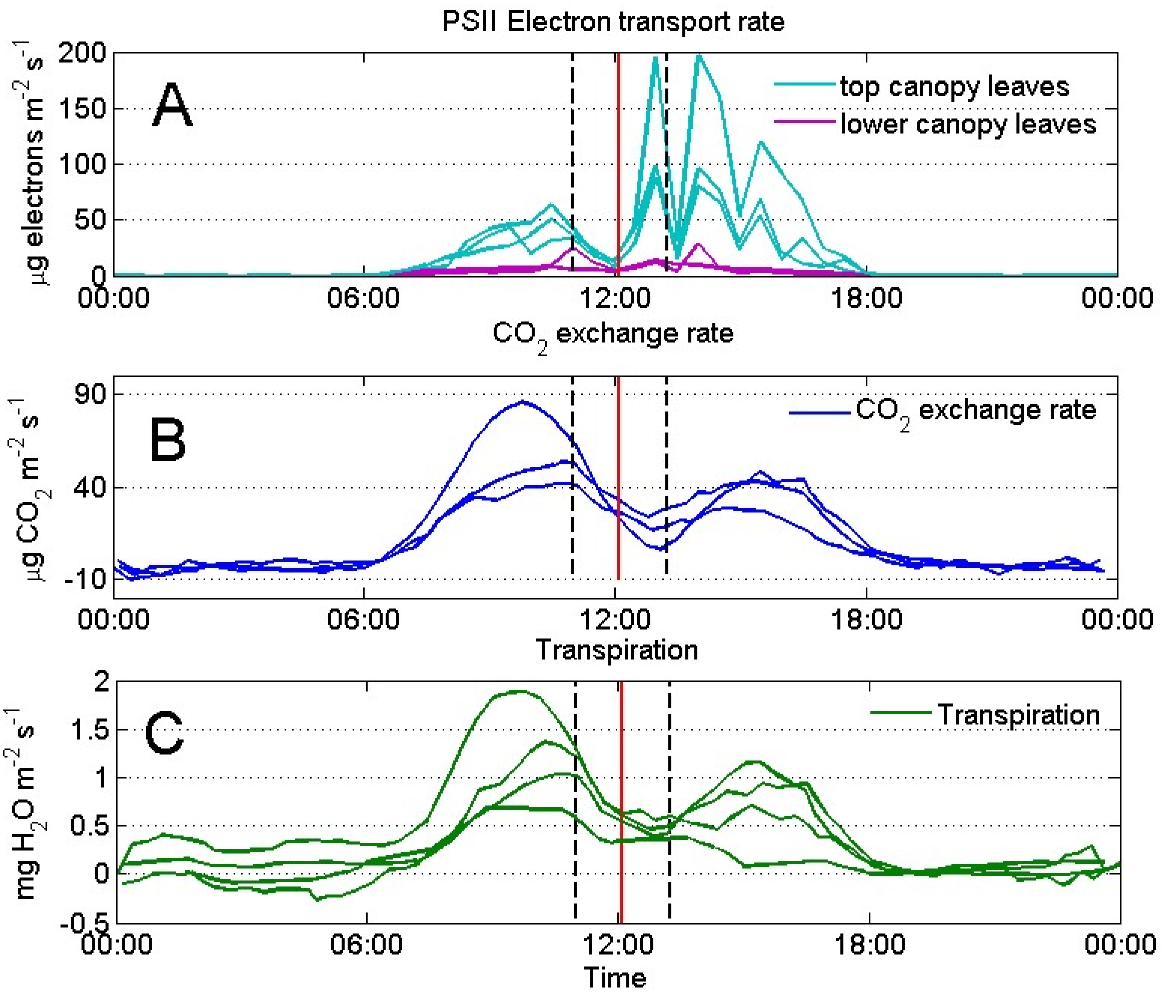

Figure S3: Dynamics of electron transport rate (ETR) of PSII in needles at five different locations within the Scots pine canopy (A). CO_2 exchange rate and transpiration of three to four top-canopy Scots pine branches (B and C). ETR is a measure of photosynthetic rate at the level of light reactions of photosynthesis. In contrast to gas exchange, ETR is not affected by leaf respiration or photorespiration, thus providing a complementary view of gross photosynthesis. The decrease in radiation during the eclipse exerted an instantaneous effect on the electron transport rate of photosystem II at all five measuring locations. Decreased ETR resulted in subsequent reduction in CO_2 assimilation which was also accompanied with a decrease in transpiration. The data reflects the strong and rapid control of photosynthesis by PAR at this temporal scale, as during the solar eclipse. Red line: maximum phase, dashed black lines: beginning and end time of the eclipse.

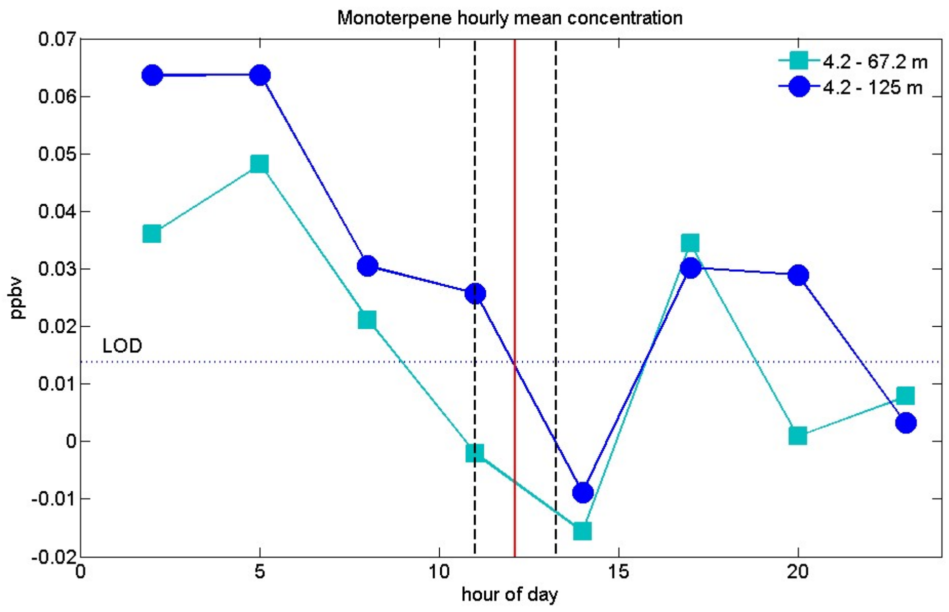

Figure S4. Hourly mean monoterpene concentration difference measured by an Proton Transfer Reaction Mass Spectrometer (PTR-MS) from different heights of the 125 m mast. Dark blue line is an average of all measured heights and light blue line depicts the five closest measurement data from ground level. The data shows expected low levels of monoterpenes and during the eclipse the concentrations decrease under the detection limit. Dashed blue LOD line represents the limit of detetion (LOD) for hourly mean measurements, LOD = 0.013 ppbv. Red line: maximum phase, dashed black lines: beginning and end time of the eclipse.

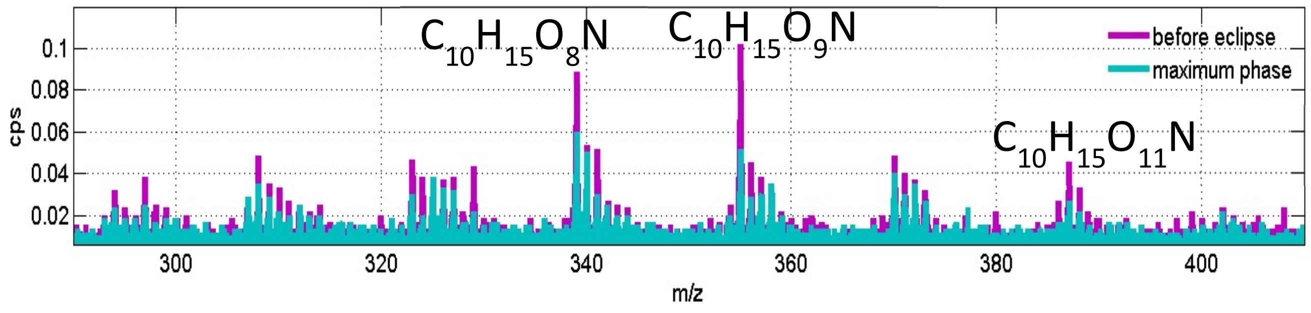

Figure S5. Spectra measured by the CI-APi-TOF before (purple) and during the maximum phase (blue) of the solar eclipse. The most abundant peaks that go through the biggest decrease during the event are depicted with chemical compositions, $C_{10}H_{15}O_8N$, 339.068 Th, $C_{10}H_{15}O_9N$, 355.063 Th and $C_{10}H_{15}O_{11}N$, 387.053 Th. All compounds are detected as clusters with the charger ion used, NO_3^-.

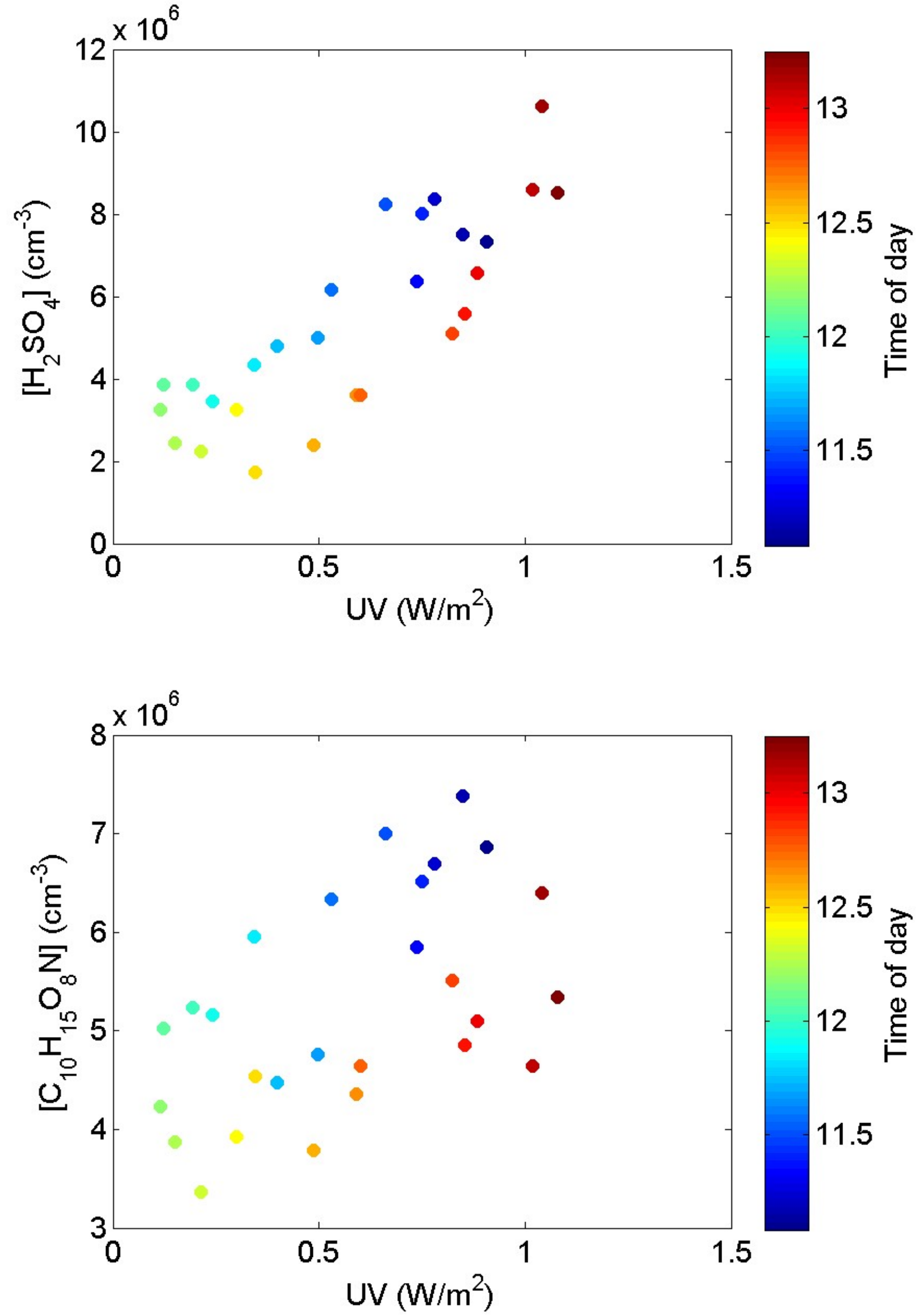

Figure S6: Concentration of sulphuric acid (H_2SO_4; top) and N-HOM ($C_{10}H_{15}O_8N$, 339 Th; bottom) as a function of UV-B on March 20, 2015. Time of day is shown in color. The maximum of eclipse was at 12:07.

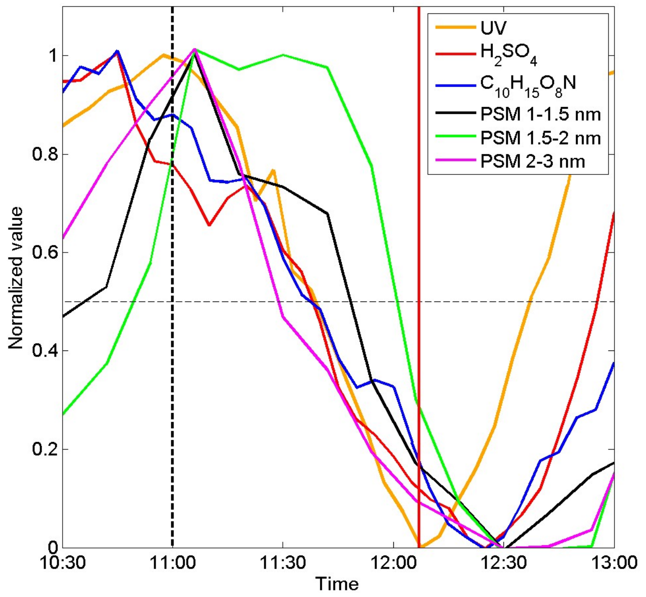

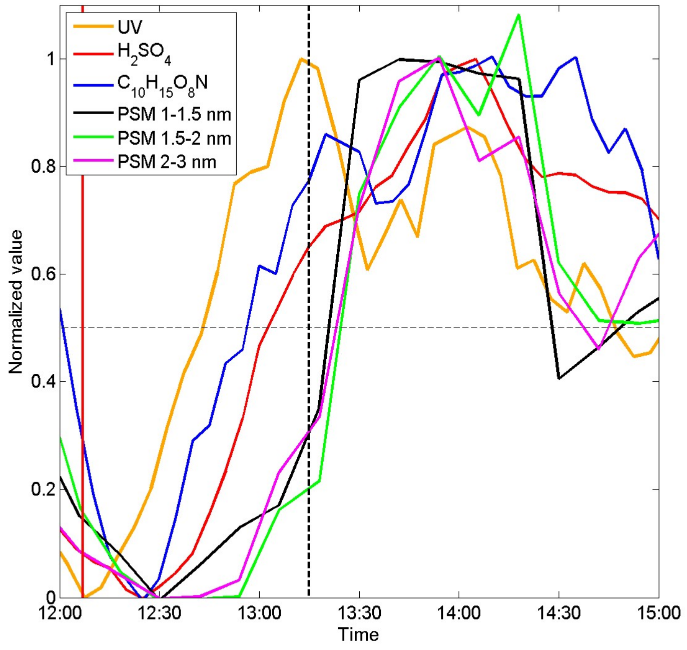

Figure S7: Normalized decline (top) and increase (bottom) of UV radiation (orange line), the concentrations of sulfuric acid (H_2SO_4; red line) and N-HOM ($C_{10}H_{15}O_8N$, 339 Th; blue line), and sub-3nm cluster concentrations measured with the PSM. The normalization is done by first subtracting the minimum value from the parameter, and then dividing the result by the difference between the maximum value and the minimum value. Vertical red line: maximum phase, vertical dashed black lines: beginning and end time of the eclipse.

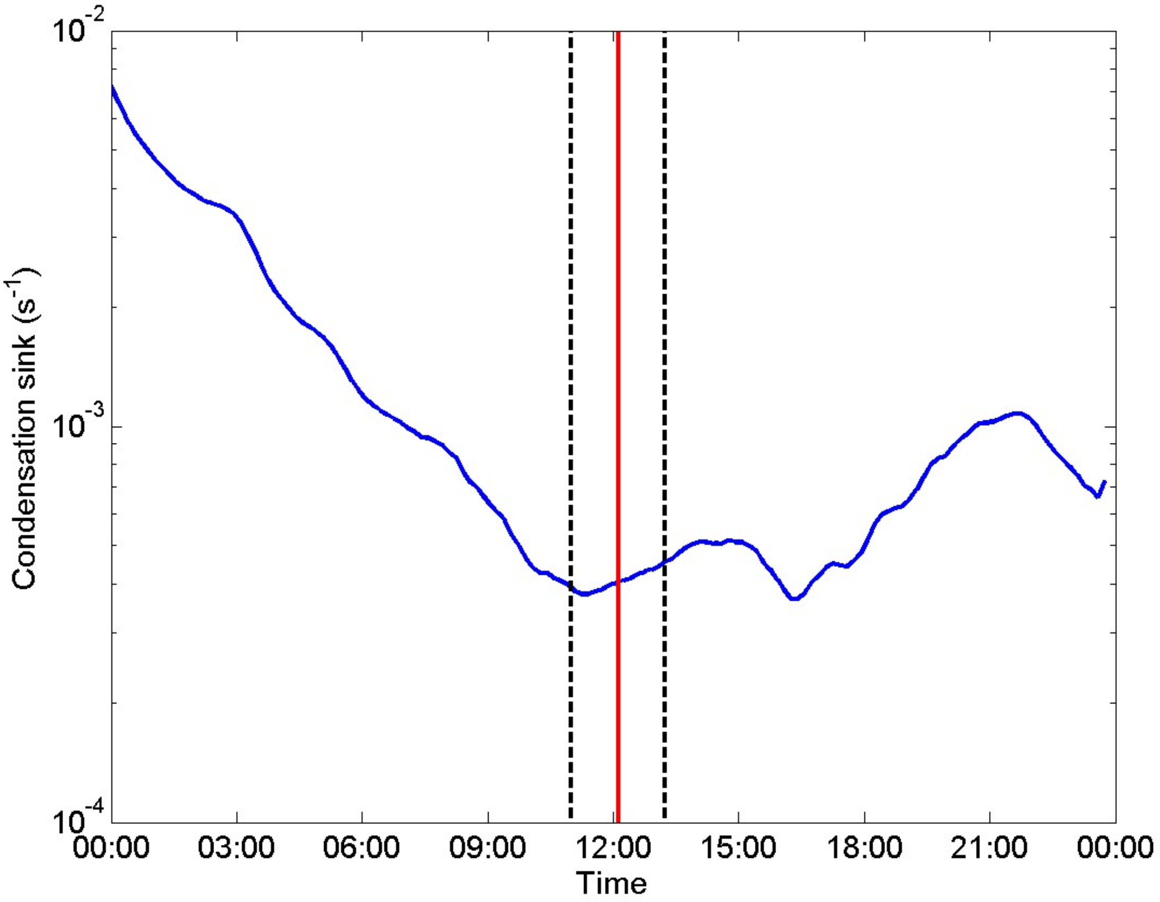

Figure S8. Condensation sink calculated from DMPS data during 20 March 2015. Red line: maximum phase, dashed black lines: beginning and end time of the eclipse.

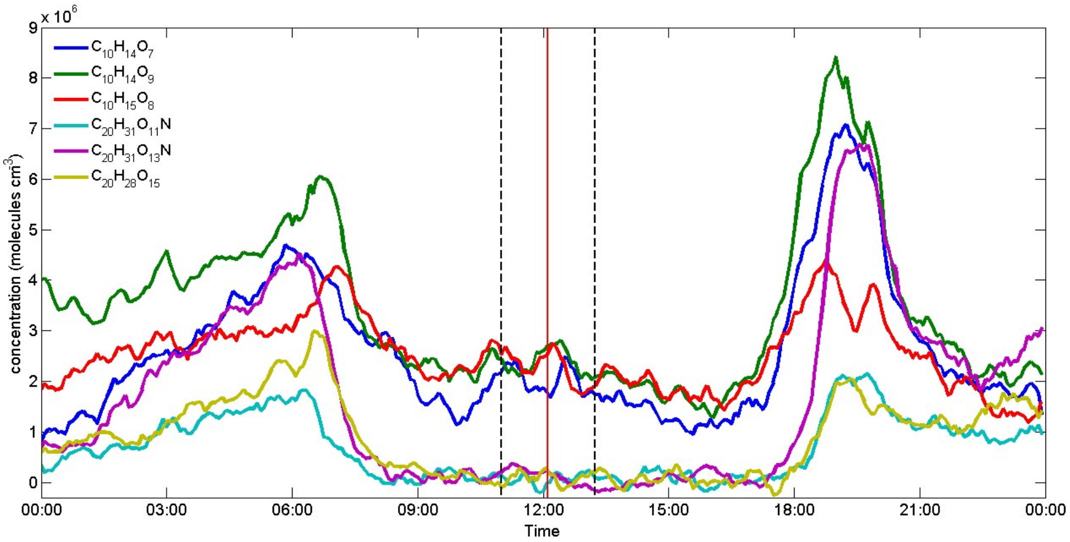

Figure S9. Concentration of HOMs during the solar eclipse day, 20 Mar 2015. The elemental compositions of the monomer peaks are: $C_{10}H_{14}O_7$ (308.062 Th), $C_{10}H_{14}O_9$ (340.052 Th) and $C_{10}H_{15}O_8$ (325.065 Th). The compounds with higher masses are suggested to have elemental compositions of $C_{20}H_{31}O_{11}N$ (523.178 Th), $C_{20}H_{31}O_{13}N$ (555.168 Th) and $C_{20}H_{28}O_{15}$ (570.131 Th). All mentioned m/z contain the mass of the charger ion, NO_3^-. Red line: maximum phase, dashed black lines: beginning and end time of the eclipse.

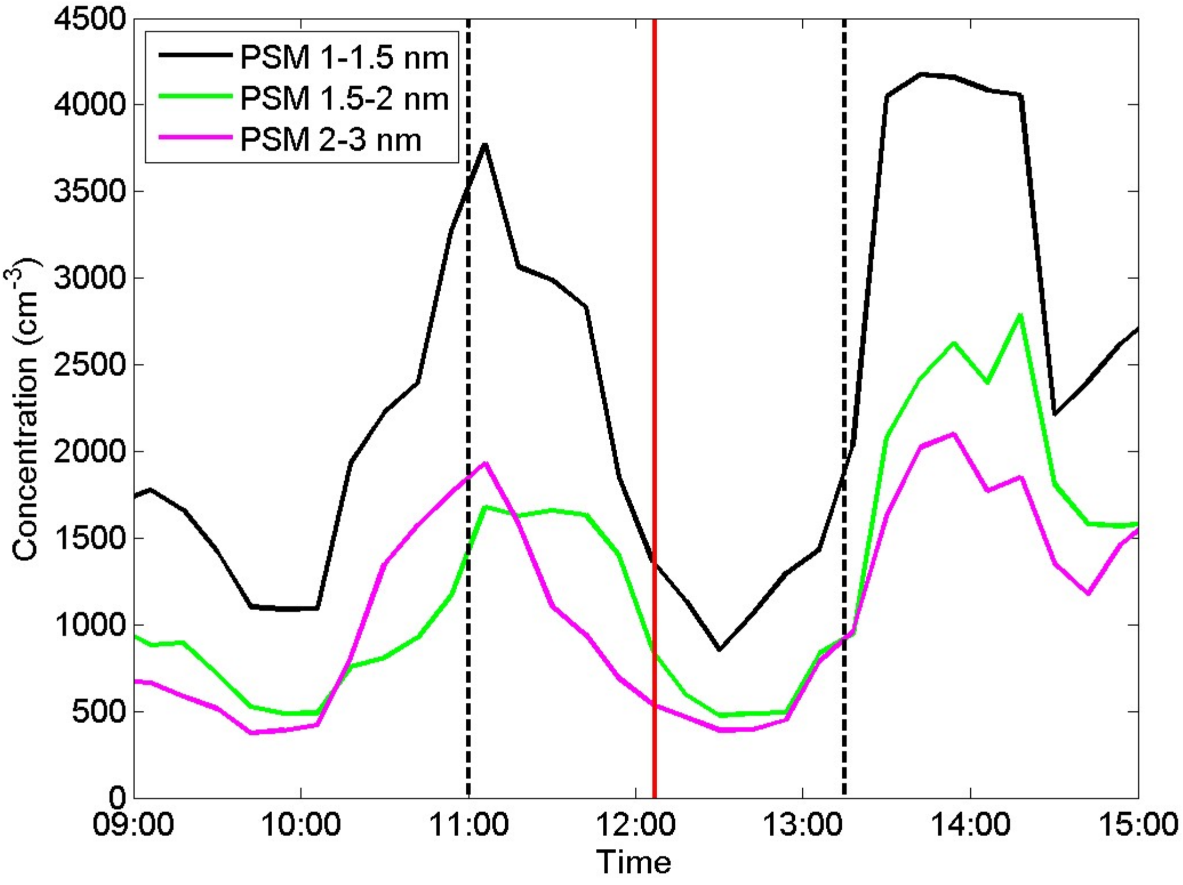

Figure S10. Concentrations of clusters and particles in different sub-3nm size bins measured with the PSM during the eclipse. Red line: maximum phase, dashed black lines: beginning and end time of the eclipse.

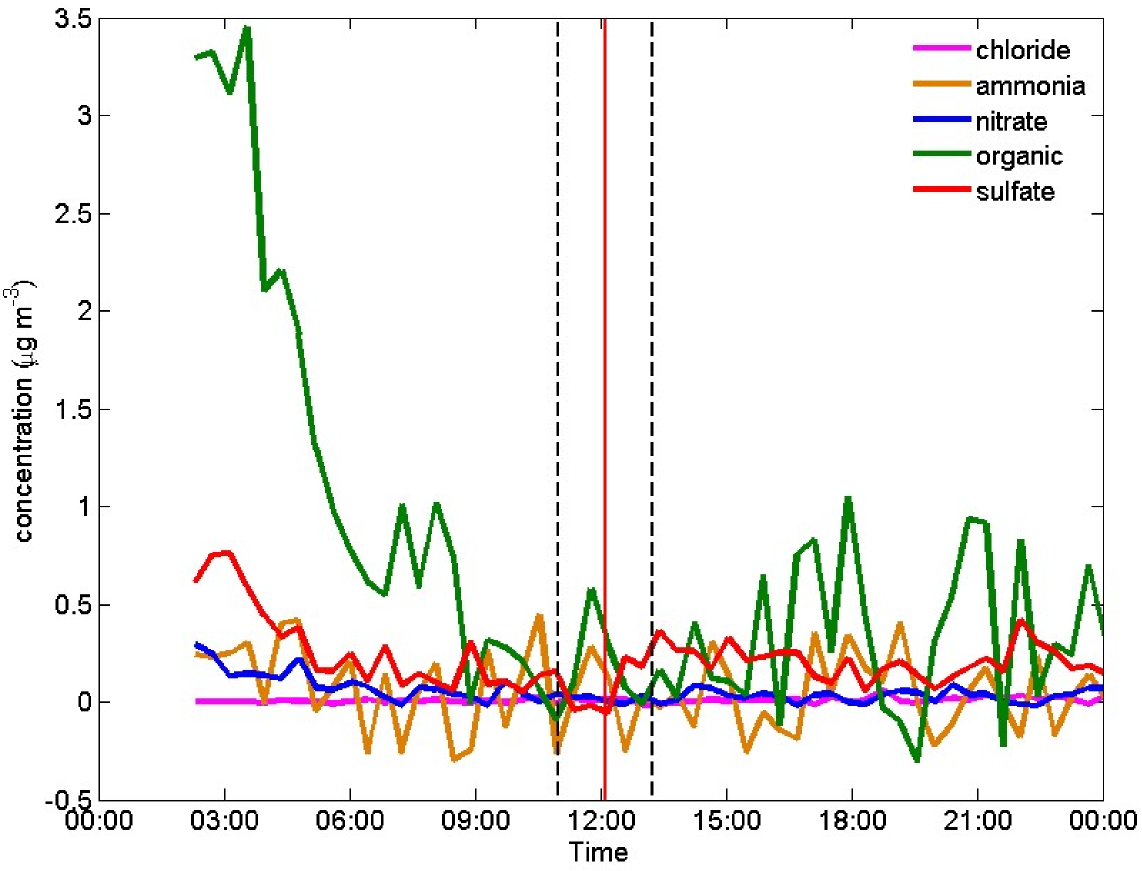

Figure S11. Measured concentration of certain chemical composition of non-refractory sub-micron aerosol particles by the Aerosol Chemical Speciation Monitor (ACSM). Red line: maximum phase, dashed black lines: beginning and end time of the eclipse.

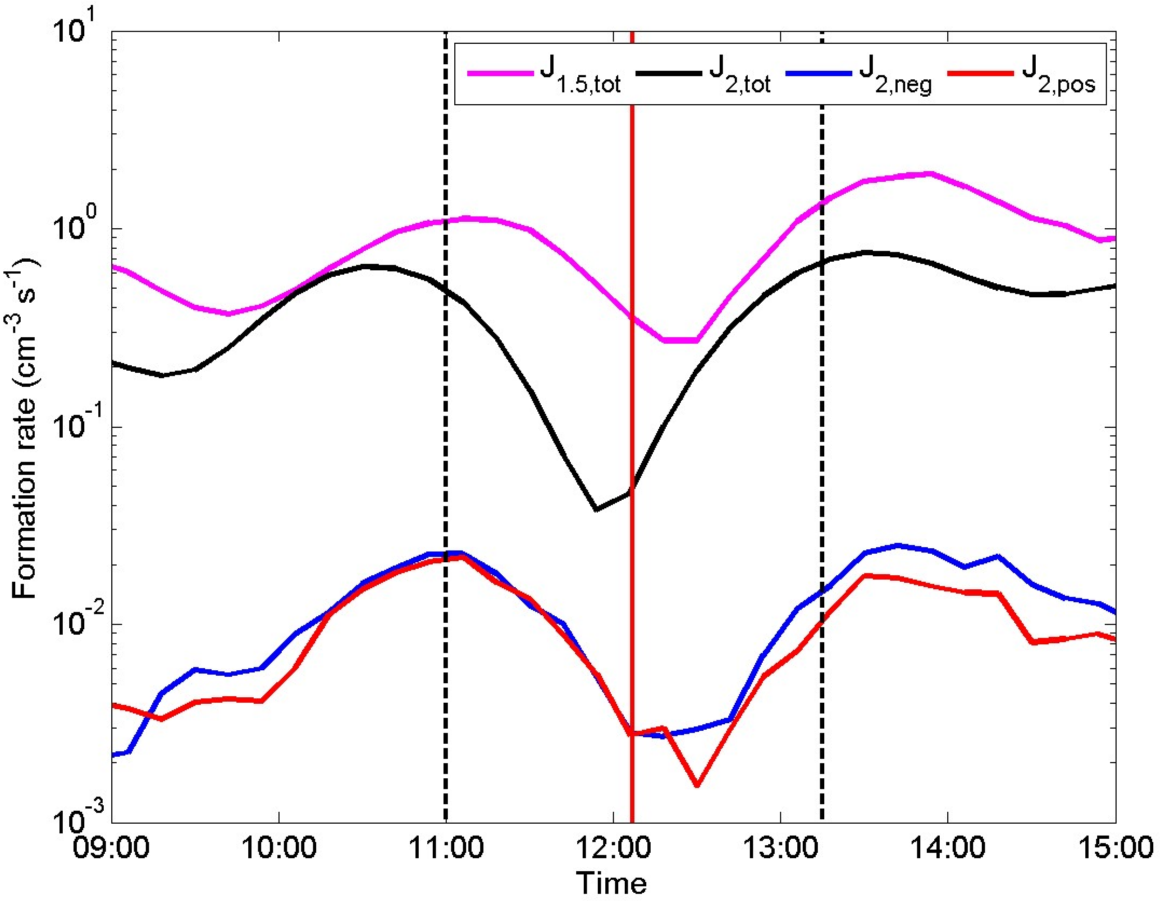

Figure S12. Formation rates of 1.5-, and 2.0-nm clusters and 2-nm negative and positive ion clusters. Red line: maximum phase, dashed black lines: beginning and end time of the eclipse.

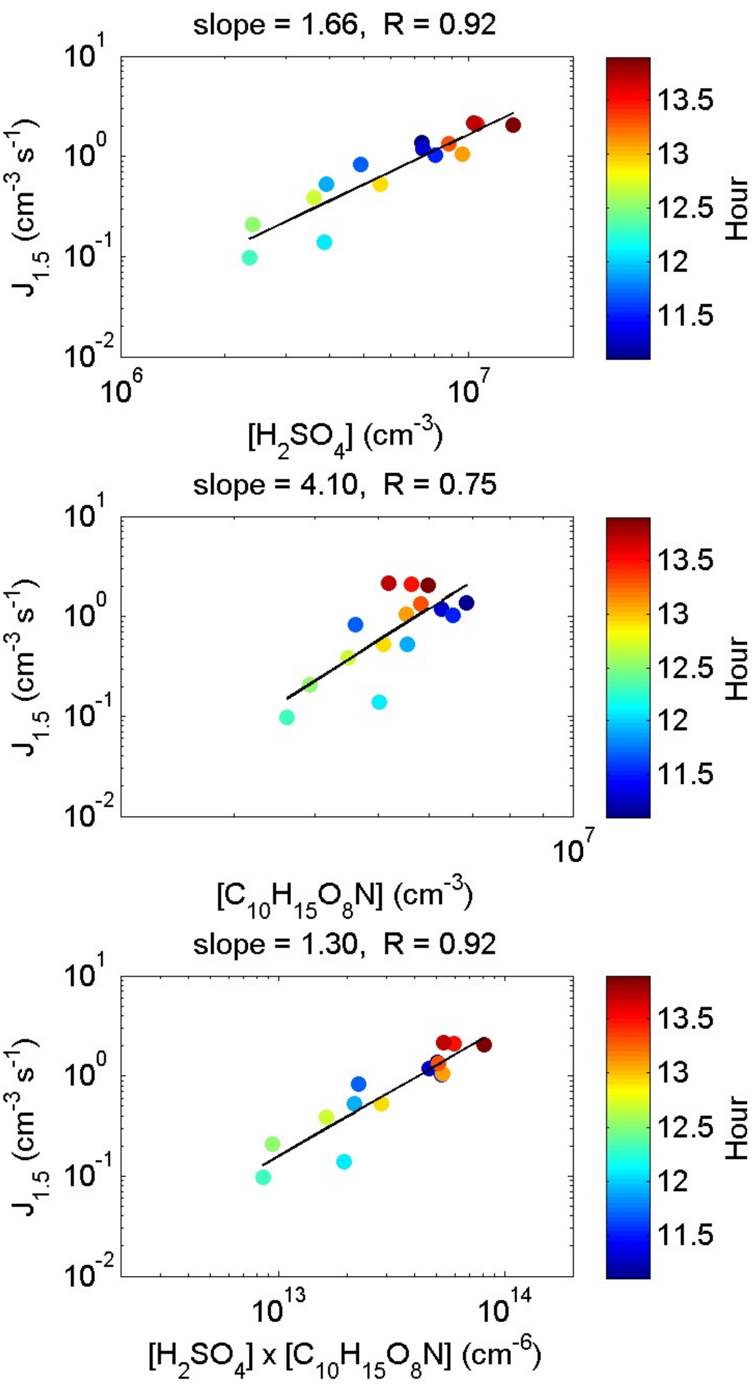

Figure S13. Formation rate of 1.5-nm clusters as a function of sulfuric acid concentration (H_2SO_4; top), N-HOM concentration ($C_{10}H_{15}O_8N$, 339 Th; middle) and their product (bottom) during eclipse.

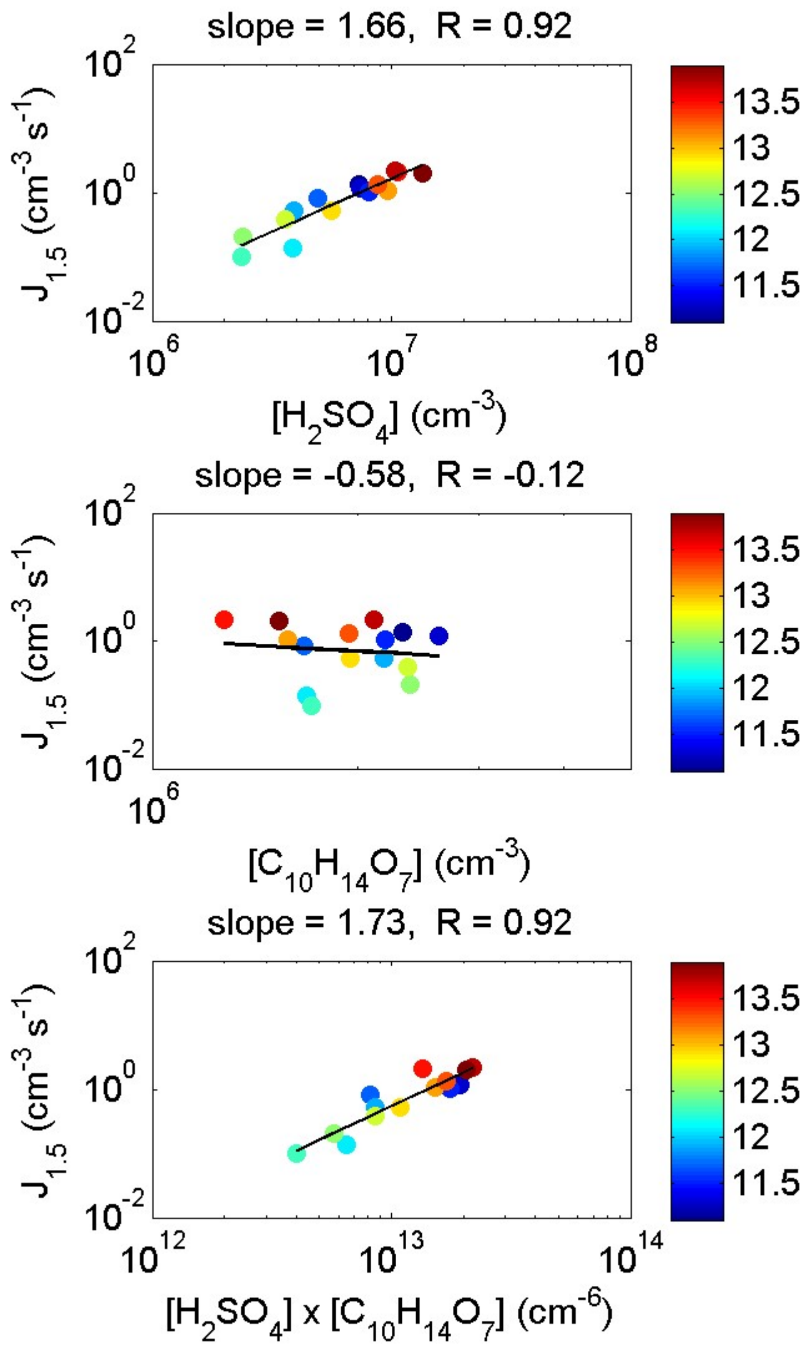

Figure S14. Formation rate of 1.5-nm clusters as a function of sulfuric acid concentration (H_2SO_4; top), HOM concentration ($C_{10}H_{14}O_7$, 308 Th ; middle) and their product (bottom) during eclipse.

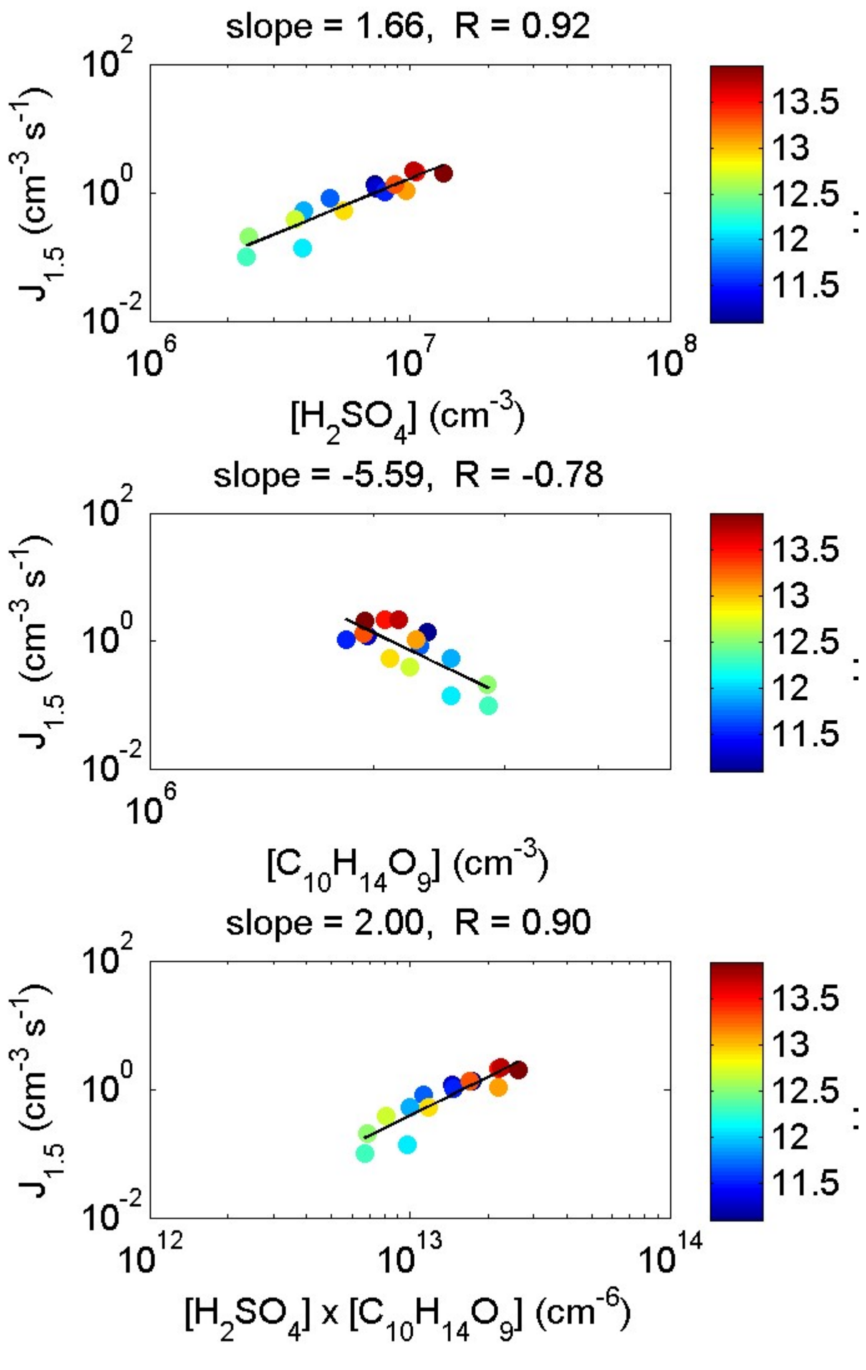

Figure S15. Formation rate of 1.5-nm clusters as a function of sulfuric acid concentration (H_2SO_4; top), HOM concentration ($C_{10}H_{15}O_9$, 340 Th; middle) and their product (bottom) during eclipse.

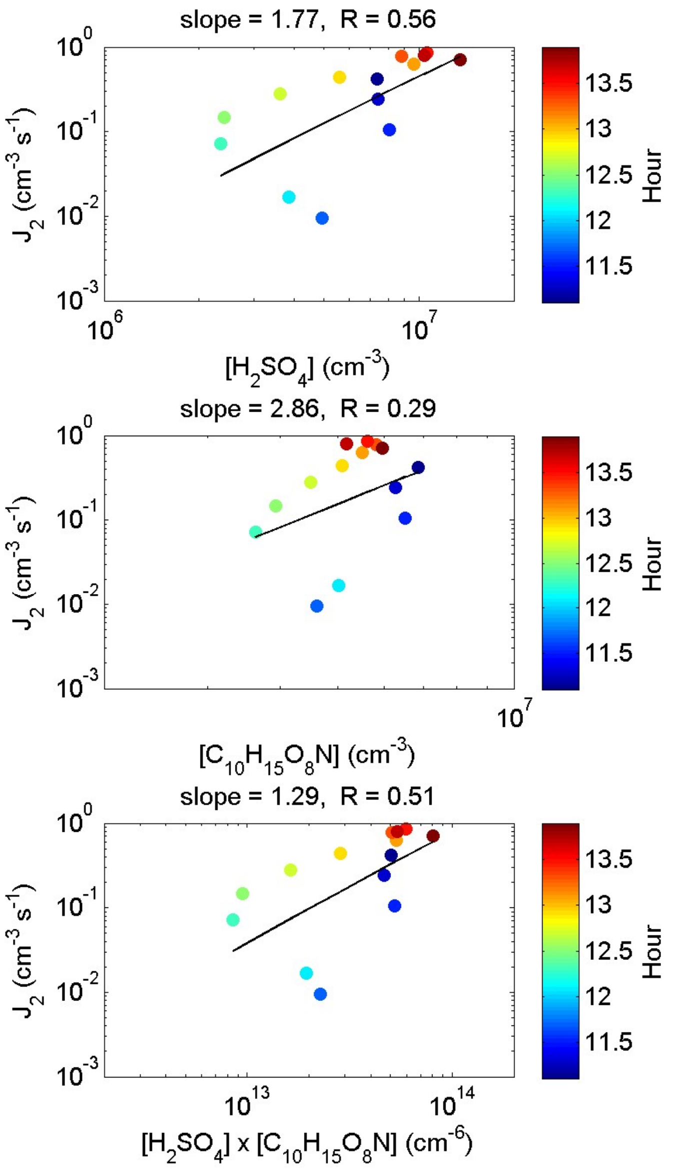

Figure S16. Formation rate of 2-nm clusters as a function of sulfuric acid concentration (H_2SO_4; top), N-HOM concentration ($C_{10}H_{15}O_8N$, 339 Th; middle) and their product (bottom) during eclipse.

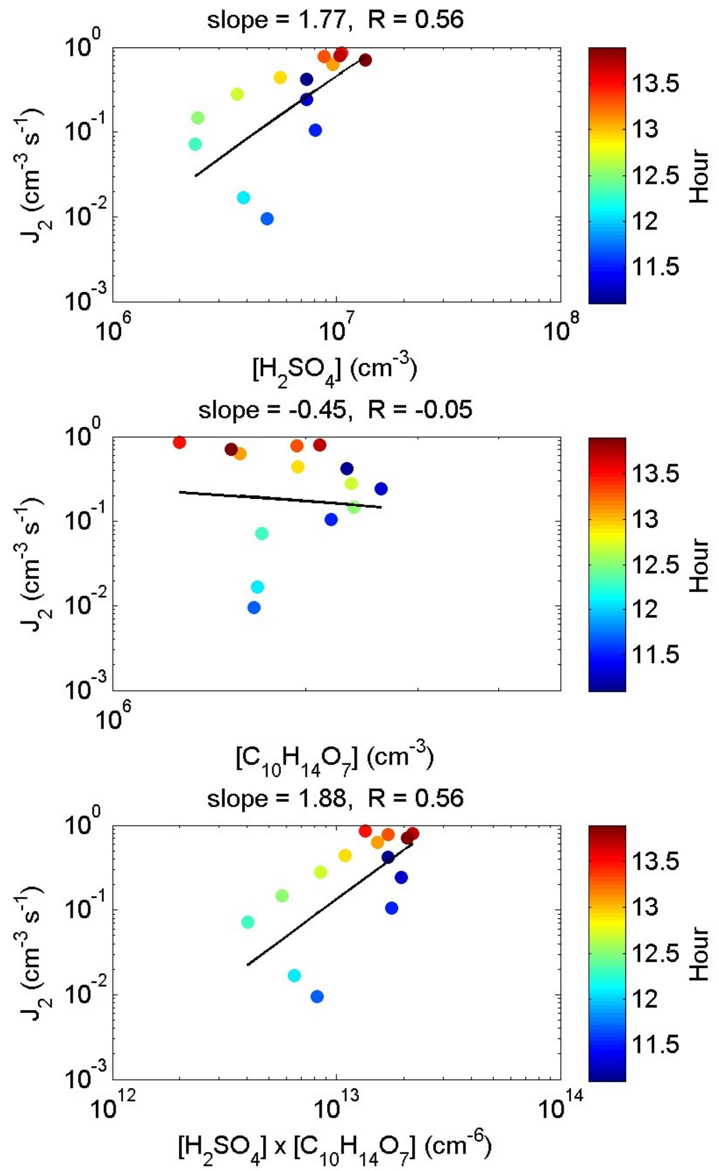

Figure S17. Formation rate of 2-nm clusters as a function of sulfuric acid concentration (H_2SO_4; top), HOM concentration ($C_{10}H_{14}O_7$, 308 Th ; middle) and their product (bottom) during eclipse.

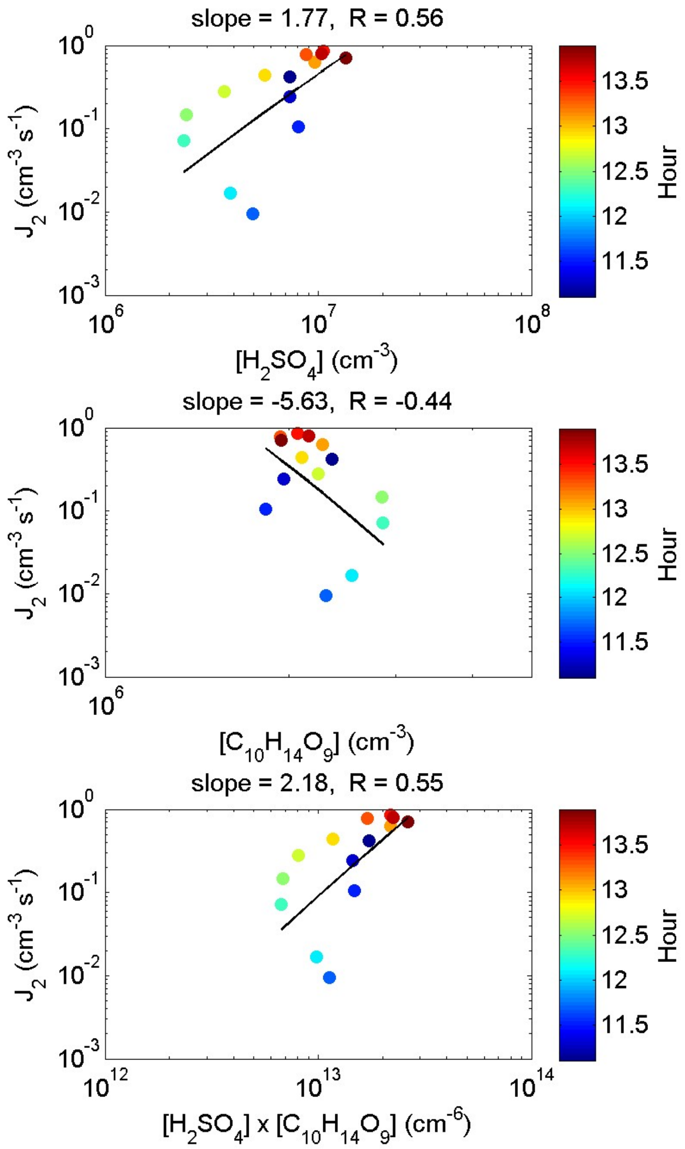

Figure S18. Formation rate of 1.5-nm clusters as a function of sulfuric acid concentration (H_2SO_4; top), HOM concentration ($C_{10}H_{15}O_9$, 340 Th; middle) and their product (bottom) during eclipse.

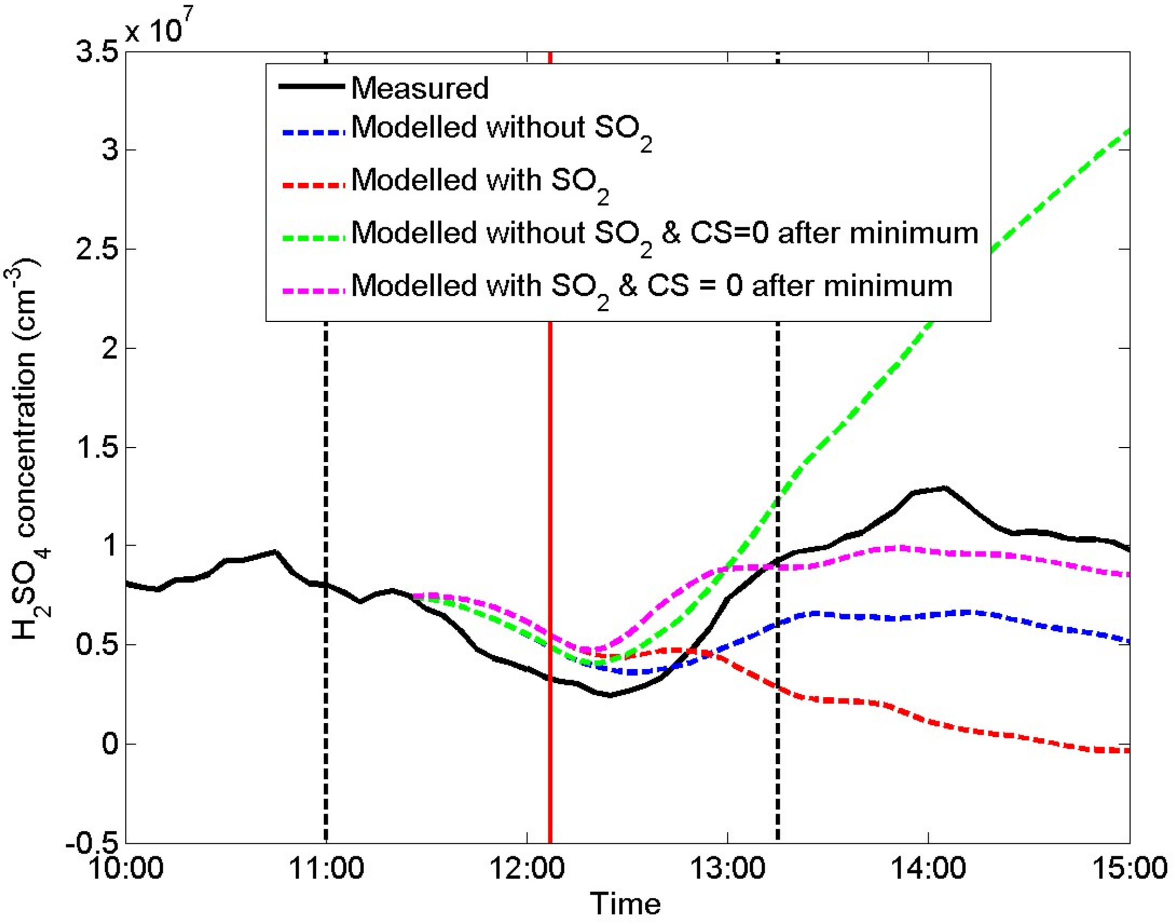

Figure S19. Measured and modelled concentration of sulfuric acid (H_2SO_4). The measured concentration is depicted with a solid black line and the modelled concentrations are shown with dashed lines. The model that does not include SO_2 is depicted with a blue line and the model including SO_2 with a red line. The model that does not include SO_2 concentration and where CS is set to zero after the minimum is shown in green, and the corresponding model including SO_2 is shown in magenta. Red line: maximum phase, dashed black lines: beginning and end time of the eclipse.

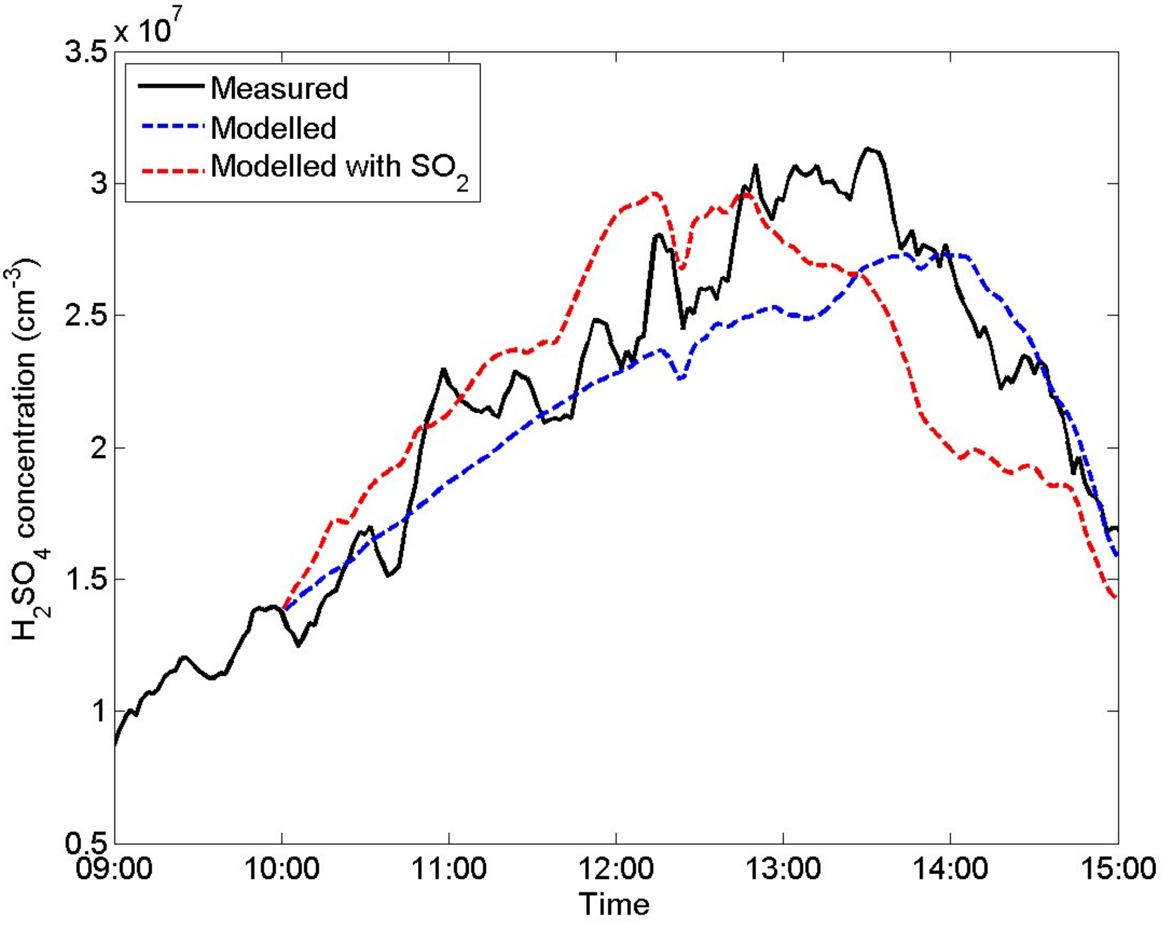

Figure S20. Measured and modelled concentration of sulfuric acid (H_2SO_4) a few days before the eclipse, 18 March 2015. The measured concentration is depicted with a solid black line. The modelled concentration obtained withouth SO_2 concentration is depicted with a blue dashed line and the modelled concentration obtained by including SO_2 in the model is depicted with a red dashed line.

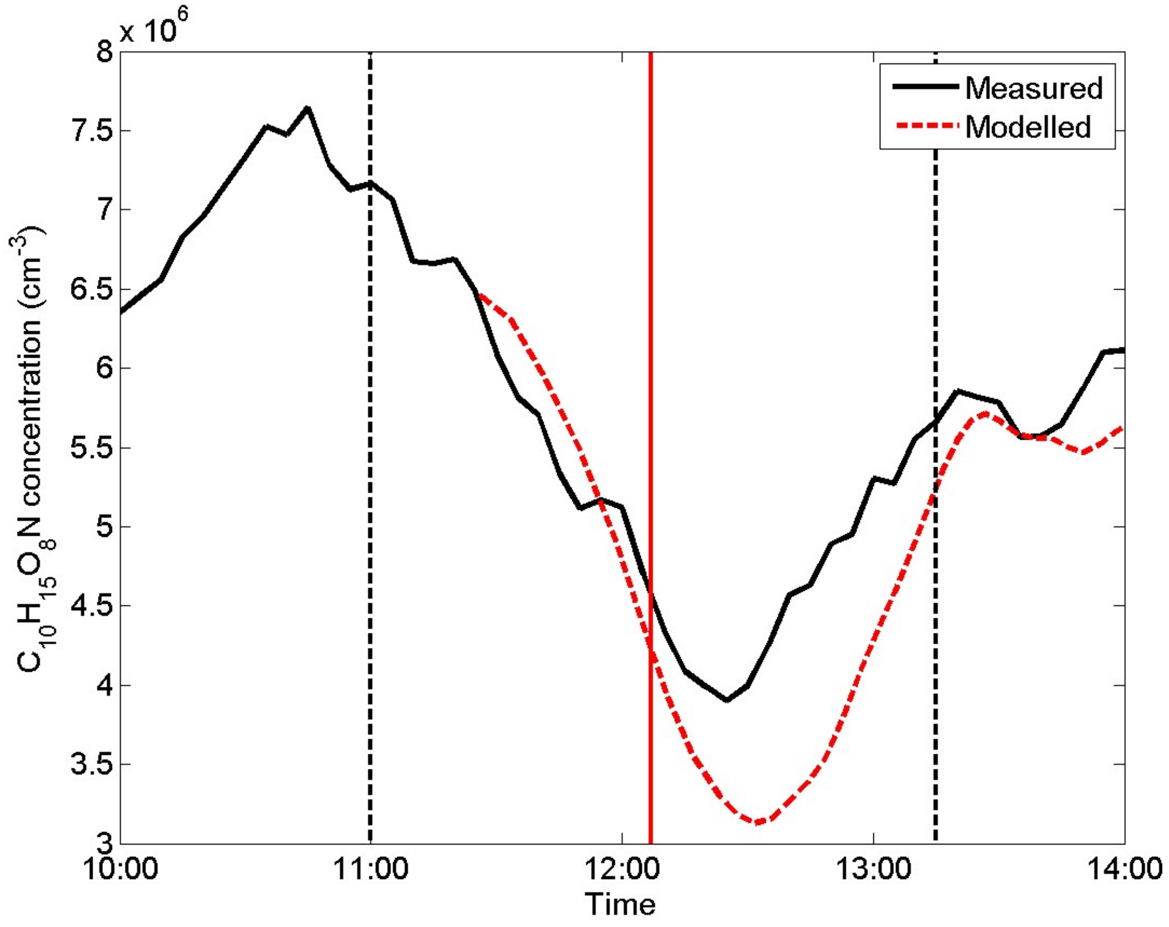

Figure S21. Measured and modelled concentration of N-HOM ($C_{10}H_{15}O_8N$, 339 Th; blue lines). The measured concentration is depicted with a solid black line and the modelled concentration with a red dashed line. Red line: maximum phase, dashed black lines: beginning and end time of the eclipse.

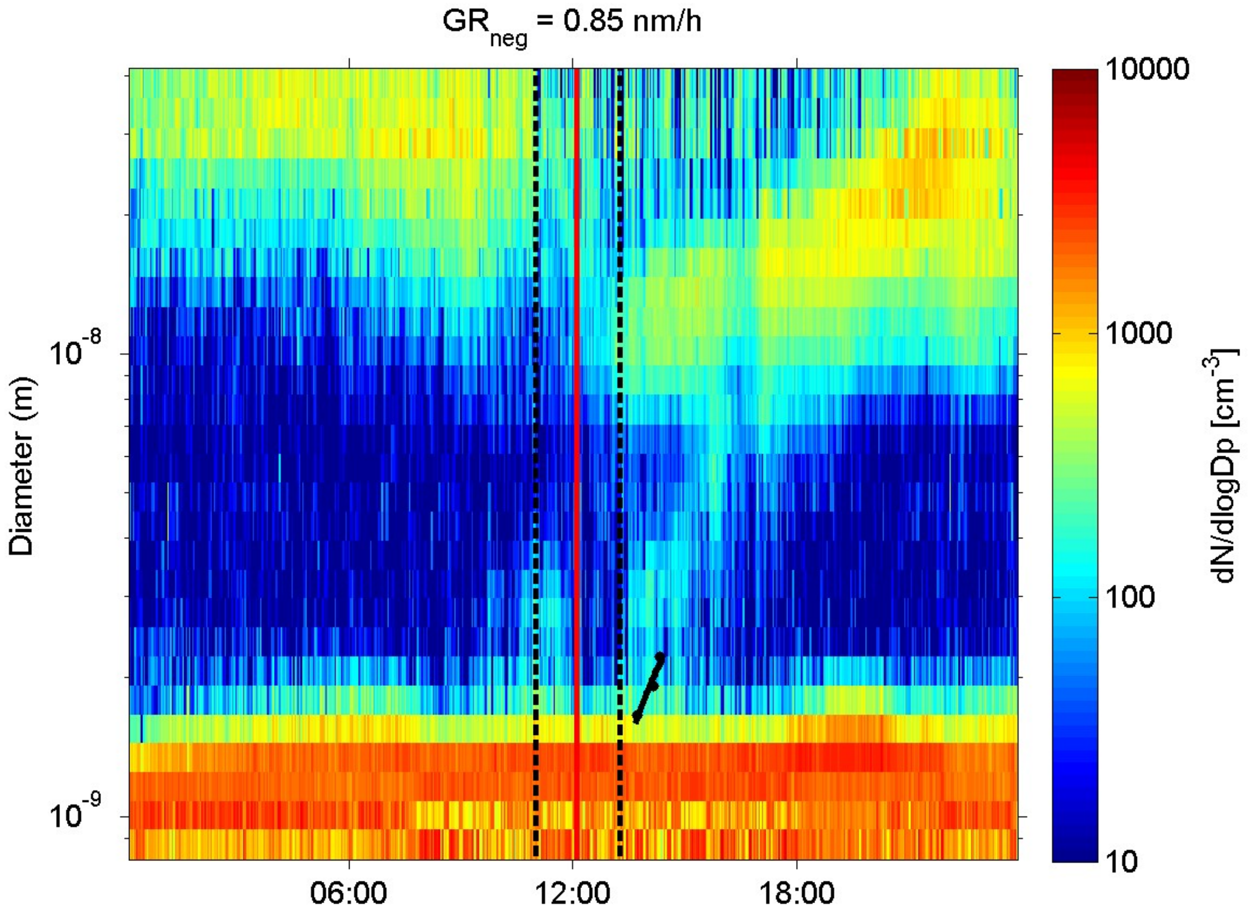

GR$_{neg}$ = 0.85 nm/h
Diameter (m)
10^{-8}
10^{-9}
06:00
12:00
18:00
10000
1000
100
10
dN/dlogDp [cm^{-3}]

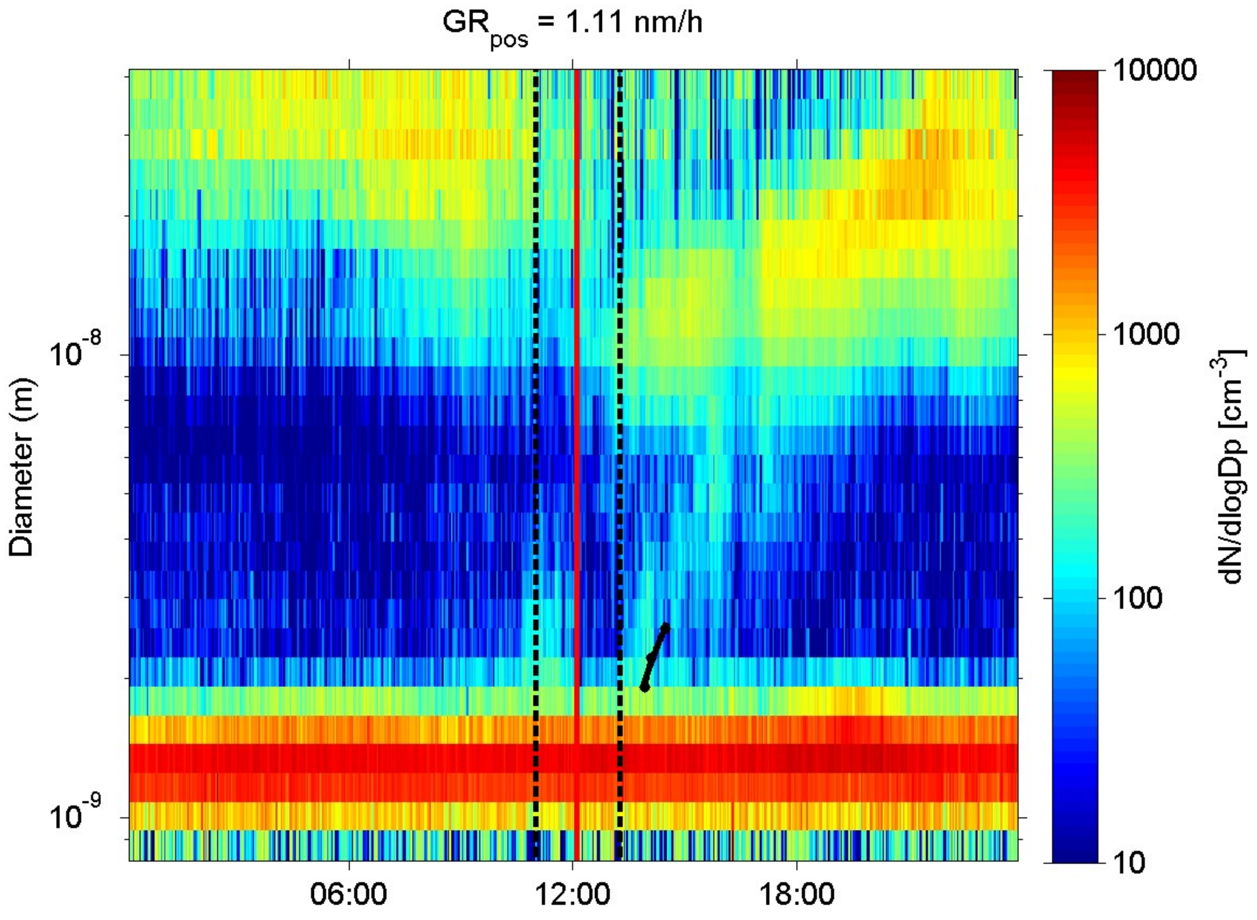

Figure S22. Growth rates of 2-3 nm negative (top panel) and positive (bottom panel) clusters determined from NAIS ion mode data. Both negative and positive ions grow around 1 nm/h (negative, 0.9 nm/h and positive 1.1 nm/h). Red line: maximum phase, dashed black lines: beginning and end time of the eclipse.
